# An Activatable Near-Infrared Chromophore for Multispectral Optoacoustic Imaging of Tumor Hypoxia and for Tumor Inhibition

**DOI:** 10.7150/thno.36755

**Published:** 2019-09-25

**Authors:** Jing Huang, Yinglong Wu, Fang Zeng, Shuizhu Wu

**Affiliations:** State Key Laboratory of Luminescent Materials & Devices, College of Materials Science & Engineering, South China University of Technology, Guangzhou 510640, China.

**Keywords:** optoacoustic, imaging, hypoxia.

## Abstract

Hypoxia is a key hallmark of solid tumors and tumor hypoxia usually contributes to cancer progression, therapeutic resistance and poor outcome. Accurately detecting and imaging tumor hypoxia with high spatial resolution would be conducive to formulating optimized treatment plan and thus achieving better patient outcome.

**Methods**: Tumor hypoxia can cleave the azo linker and release a NIR fluorophore (NR-NH_2_) and release the active drug as well. NR-NH_2_ shows a strong absorption band at around 680 nm and a strong fluorescence band at 710 nm, allowing for both multispectral optoacoustic tomography imaging (MSOT) and fluorescent imaging of tumor hypoxia in a tumor-bearing mouse model.

**Results**: Liposome encapsulated with the activatable chromophore (NR-azo) for detecting/imaging tumor hypoxia and for tumor inhibition was demonstrated. For this chromophore, a xanthene-based NIR fluorophore acts as the optoacoustic and fluorescent reporter, an azo linker serves as the hypoxia-responsive moiety and a nitrogen mustard as the therapeutic drug. NR-azo shows an absorption at around 575 nm but exhibits negligible fluorescence due to the existence of the strong electron-withdrawing azo linker.

**Conclusion**: We demonstrated an optoacoustic and fluorescent system for not only imaging tumor hypoxia in vivo but also achieving tumor inhibition.

## Introduction

Optical imaging modalities such as fluorescent imaging have been extensively investigated for detection and imaging because of its advantages including high sensitivity; 1-12 but the pure optical imaging methods have such limitations as low spatial resolution with unsatisfactory penetration depth due to high-degree light scattering by tissue. 13-15 On the other hand, as a newly emerged imaging modality, the optoacoustic (photoacoustic) imaging can overcome these inherent drawbacks of fluorescent imaging. 16-31 By detecting the ultrasonic signal generated by local tissue's thermoelastic expansion resulted from absorption of the external excitation laser, optoacoustic imaging can integrate the advantages of pure optical imaging with those of acoustic imaging, 32-37 which endows the optoacoustic imaging with higher spatial resolution and larger penetration depth. 38-43 Multispectral optoacoustic tomography (MSOT) is an optoacoustic imaging technique which can distinguish ultrasound signals generated by different agents. It separates the signals of an exogenous contrast agent from the background signals of endogenous substances (such as hemoglobin and melanin) through irradiating the sample with multiple wavelengths and then detecting ultrasound waves, followed by image reconstruction and multispectral unmixing. 16,17,25,44-46 As a result, a specific photoabsorber can be separately visualized in MSOT images. 16,17,25,44-46 More importantly, via rendering stacks of cross-sectional (tomographic) images as the maximum intensity projection images, the orthogonal-view three-dimensional (3D) MSOT images can be obtained, which enable us to precisely locate the disease focus through detecting biomarkers. 25,44-46

In solid tumors, hypoxia arises as a result of the uncontrolled oncogene-driven proliferation of cancer cells, the altered metabolic features as well as the abnormal tumor blood vessels without an efficient vascular network; and it has been considered as a key hallmark of solid tumors. 47-53 It is known that tumor hypoxia is correlated with poor prognosis for cancer patients, due to its association with aggressive tumor phenotype and therapeutic resistance. 48,49 Hence, the detection of tumor hypoxia plays a pivotal role in developing appropriate treatment strategies for solid tumors. 50-53

For non-invasive detection of hypoxia, some optical probes have been developed with nitroaryl or quinone group as recognition moiety through responding to some intracellular reductases which are overexpressed in hypoxic tumor environment. 54-75 For example, Nagano et al. and other groups developed azo-based fluorescent probes for detecting hypoxia; 62-73 and Kim's group and others went further and designed some fluorescent systems which could not only detect tumor hypoxia but also trigger the release of drug. 74,75

On the other hand, optoacoustic techniques have been employed to detect hypoxia by imaging endogenous contrast agents such as hemoglobin. 76,77 Up to now only a few activatable optoacoustic probes have been reported for detecting tumor hypoxia by Chan and coworkers, 78,79 who designed optoacoustic probes for hypoxia based on one-electron reductions performed by nitroreductases. Despite the advances obtained so far in the optoacoustic detection of tumor hypoxia, there remains much room for improvement, especially in terms of the design of activatable system for simultaneous tumor hypoxia imaging and hypoxia-induced release of active anti-tumor drug, as well as the mapping of the biodistribution and metabolism of the probe/drug system by tomographic technique.

**Scheme 1:**
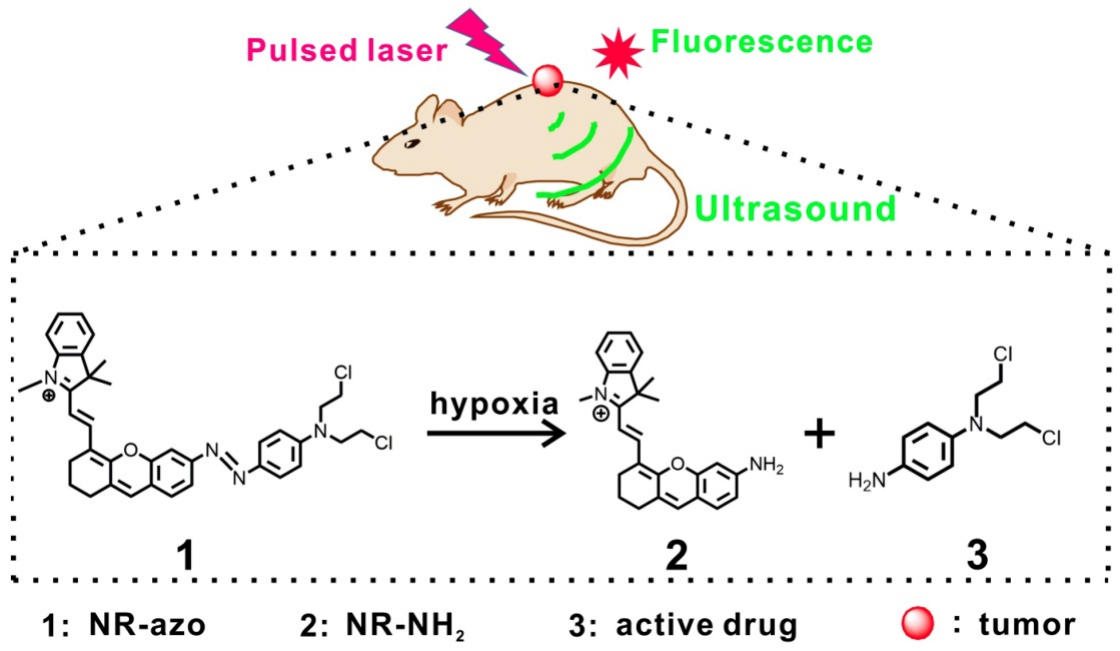
Schematic illustration for the chromophore's (NR-azo) response toward hypoxia in tumor-bearing mice model.

Taking into consideration the above situation, we herein demonstrate an activatable chromophore (NR-azo) as a dual-purpose system that can serve as an optoacoustic probe for 3D tumor hypoxia imaging and a hypoxia-responsive prodrug. In this chromophore system, a xanthene chromophore acts as the reporter, an azo linker serves as the hypoxia-responsive moiety and a nitrogen mustard as the therapeutic drug. Notably, the severe side effects (toxicity) of the anti-cancer drug nitrogen mustard 15,80 could be greatly reduced after being incorporated into the system, since the active drug will only be released under hypoxia. The dual-purpose system NR-azo exhibits an absorption band at around 575 nm but shows negligible fluorescence due to its covalent connection to the azo group. When the azo linker is cleaved under hypoxia, a chromophore (NR-NH_2_) is generated with red-shifted absorption and the active drug nitrogen mustard is released as well. NR-NH_2_ shows prominent absorption at around 680 nm and strong fluorescence at about 710 nm, which makes it an ideal exogenous activatable contrast agent for MSOT and fluorescent imaging of tumor hypoxia. In the meantime, the released drug can be employed for tumor inhibition. The schematic illustration for the action of this dual-function system is displayed in Scheme 1. Our experimental results indicate that, the system responds quickly to tumor hypoxia and achieve substantial tumor inhibition, and this process can be tracked by MSOT and fluorescent imaging.

## Results and Discussion

### NR-azo's spectral properties and response toward hypoxia in vitro

**Figure 1:**
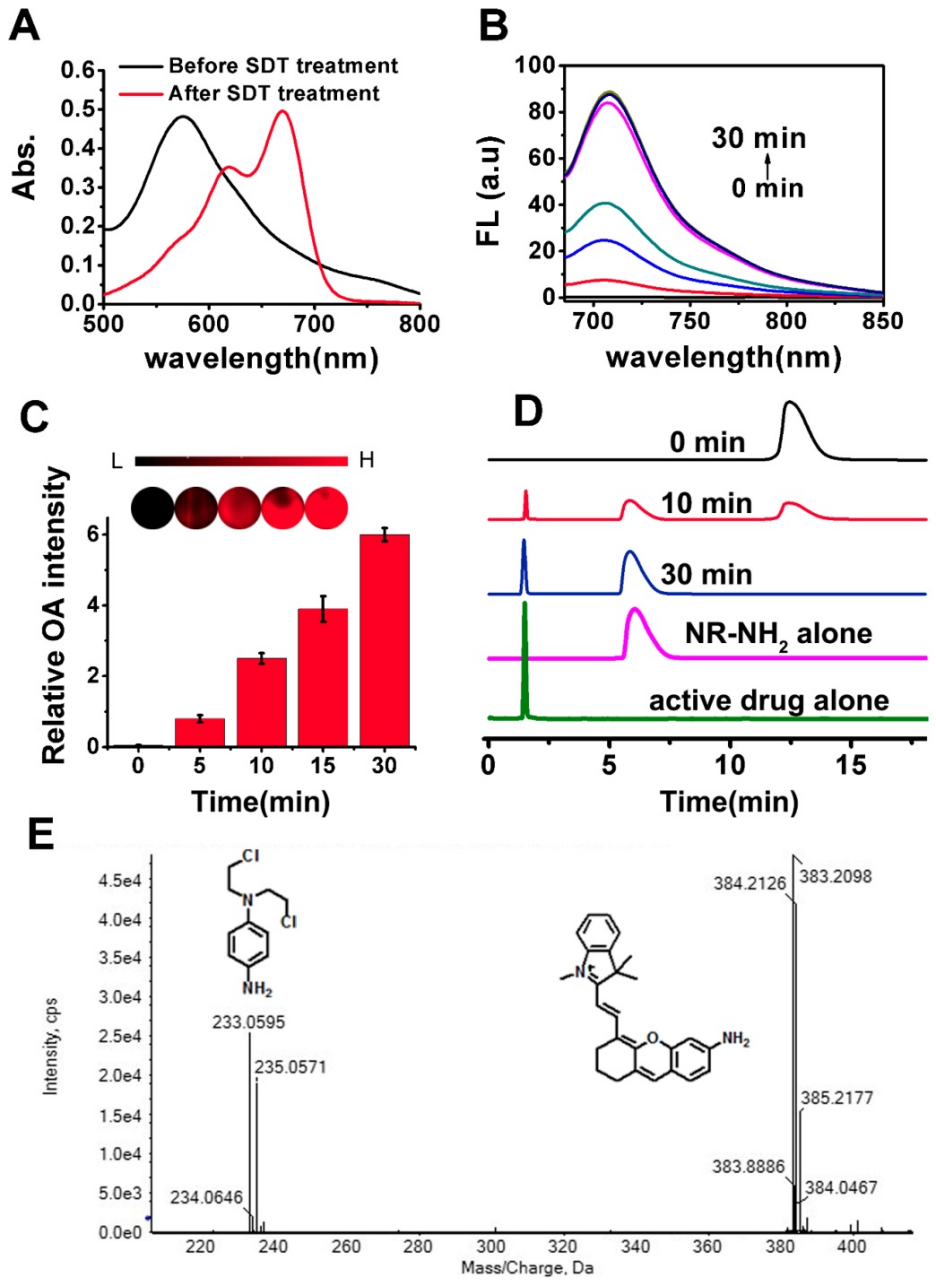
(A) Absorption spectra of NR-azo before and after treatment with SDT (100 μM). (B) Fluorescence spectra of NR-azo before and after treatment with MLM (50 μg/mL) and NADPH (cofactor for reductases, 100 μM) for different time (up to 30 minutes). (C) Optoacoustic response of NR-azo toward hypoxia (upon treatment with MLM). (D) HPLC profile for pure NR-NH_2_ (Magenta line), the active drug (green line) and NR-azo upon treated with MLM for varied time. (E) HR-MS spectrum of NR-azo upon treatment with MLM.

For synthesis of NR-azo, as shown in Scheme S1, an amino-containing xanthene fluorophore (Compound 2, NR-NH_2_) was first synthesized and then coupled with a nitrogen mustard through an azo linker to afford the final product. The intermediate compounds and the final product NR-azo were characterized by ^1^H and ^13^C NMR and mass spectroscopy, as shown in Fig. S1-S9. The absorption spectra of NR-azo and NR-NH_2_ were shown in Fig. S12A. NR-NH_2_ shows a maximum absorption at round 680 nm; while after its coupling with the nitrogen mustard through the azo linker, the resultant chromophore NR-azo exhibits a blue-shifted absorption band at about 575 nm, which is caused by the electron-withdrawing effect of the azo moiety. As shown in Fig. S12B, upon excitation at 680 nm, NR-NH_2_ displayed a fluorescence band at around 710 nm while NR-azo showed no fluorescence because of the fluorescence quenching by azo moiety. Under hypoxic conditions, intracellular reductases including azoreductase, are overexpressed in hypoxic tumor cells, which can cause the cleavage of azo groups. 74,75,81 Since sodium dithionite (SDT) can effectively and reductively cleave azo group, it is commonly used as the azoreductase mimic to evaluate the hypoxia-induced reduction and cleavage of azo. 73-75 In this study, we first investigated the response of NR-azo towards STD in vitro, and the time-dependent fluorescence spectra were recorded. As shown in Fig. S13A, for NR-azo solution, after 30 minutes of SDT treatment, strong fluorescence was observed at around 710 nm. The absorption spectra of NR-azo before and after treatment with SDT are shown in Fig. 1A. Upon SDT treatment, the absorption red-shifted to around 680 nm. By comparison (Fig. 1A and Fig. S12A), it is clear that the two spectra are quite similar to each other, indicating that the SDT treatment induces the generation of NR-NH_2_. To further confirm that the photophysical changes (absorption and fluorescence) are caused by the generation of NR-NH_2_ upon azo cleavage, we synthesized a control chromophore (NR-CLB) which contains no azo linker. The characterizations of this control are shown in Fig. S10-S11, and its absorption and fluorescence spectra before and after incubation with SDT are shown in Fig. S14. It is clear that the control chromophore showed absorption at around 570 nm, and upon excitation at 680 nm no fluorescence can be observed. Moreover, SDT treatment has no effects on the absorption or fluorescence of the control chromophore. The results support that azo cleavage play a key role in the generation of the fluorophore NR-NH_2_.

Next, mouse liver microsomes (MLM) which contains a variety of reductases including azoreductase 60,82-84 were used to examine the response of the chromophore NR-azo toward hypoxia. As shown in Fig. 1B, upon treatment with MLM (50 μg/mL) and NADPH (cofactor for reductases, 100 μM) for 30 minutes, evident increase of fluorescence intensity at around 710 nm was observed (Fig. 1B). The chromophore's optoacoustic response toward MLM was evaluated in phantom. Upon incubation with MLM, the relative optoacoustic intensities for the generated NR-NH_2_ are given in Fig. 1C. It can be seen that the relative optoacoustic intensity increases with the incubation time. These prominent spectral changes were resulted from the structural transformation from NR-azo to NR-NH_2_ due to the cleavage of azo linker. The above results indicate that the chromophore NR-azo is a promising system to serve as the hypoxia probe in fluorescent and optoacoustic detection.

To confirm the release of NR-NH_2_ from NR-azo, high performance liquid chromatography (HPLC) and high-resolution mass spectroscopy were performed. As shown in Fig. 1D, the peak of retention time at 12.5 min corresponds to NR-azo and that at 6.1 min corresponds to NR-NH_2_, while the retention time of the active drug was at 1.7 min. On the other hand, for the NR-azo upon treatment with MLM, the peak at 12.5 min reduced; and new peaks at 1.7 min and 6.1 min emerged, which well matched the peaks of the active drug and NR-NH_2_ respectively. Moreover, the release of NR-NH_2_ and the active drug nitrogen mustard was further verified by high resolution MS analysis (Fig. 1E). The peaks at m/z around 383.2098 correspond to NR-NH_2_, while the peaks at m/z around 233.0595 correspond to the active drug. These results indicate that the fluorescent NR-NH_2_ and the active drug can be simultaneously released from NR-azo upon treatment with MLM, and the fluorescent signal of NR-NH_2_ can be used to trace the release of the anti-tumor drug. To test the selectivity of NR-azo, we incubated it with SDT and some biologically-relevant potential interferents (such as metal ions, common reductants in physiological environment and some enzymes) respectively for 30 min under nitrogen atmosphere or ambient atmosphere (containing ca. 21% O_2_) and recorded their fluorescence intensities. The results are presented in Fig. S15. For NR-azo, whether under nitrogen atmosphere or normal atmosphere, only treatment with SDT could greatly enhance its fluorescence. While for the other potential interferents, very low fluorescence intensities were observed under the same conditions. The results indicate that NR-azo won't be affected by these potential interferents.

**Figure 2:**
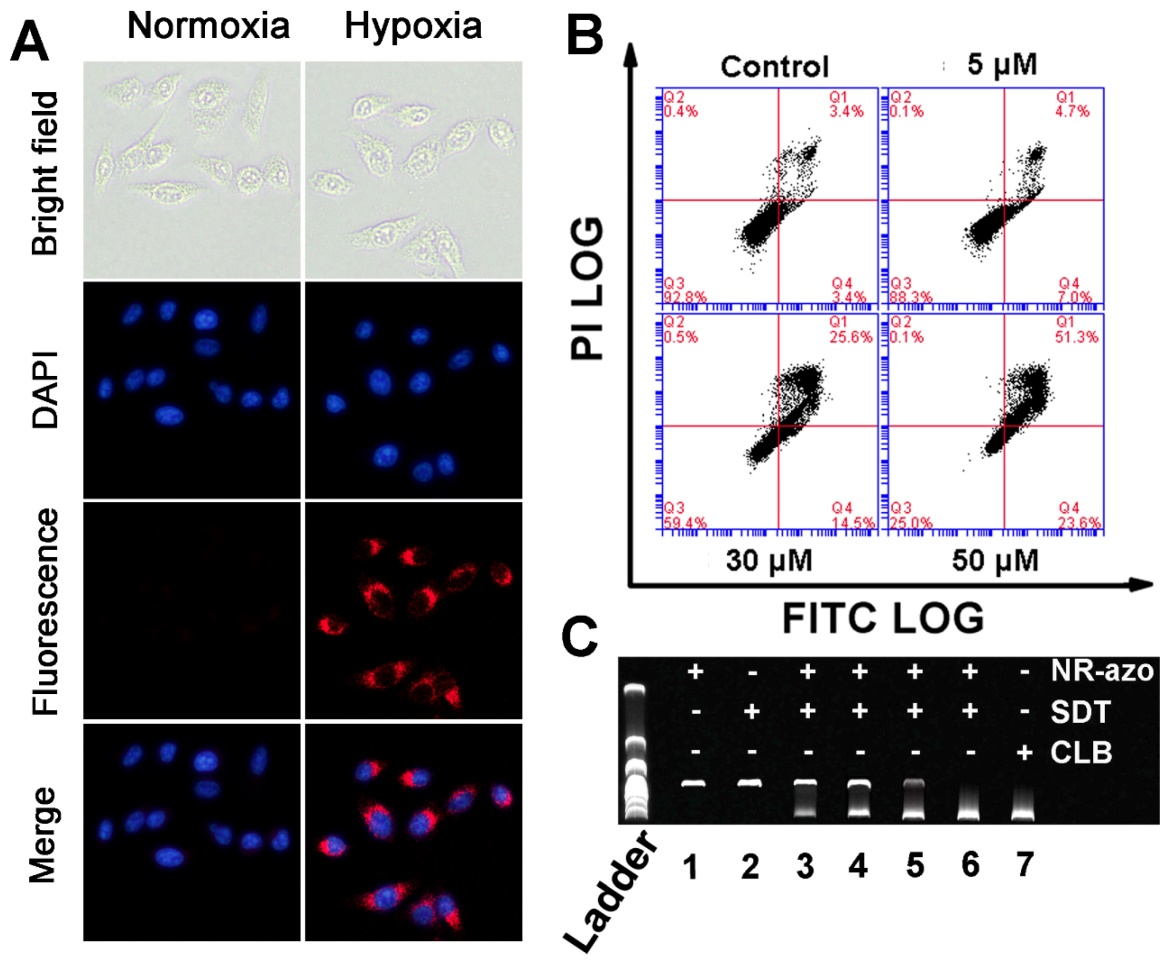
(A) Fluorescence images for HepG2 cells incubated with 10 μM NR-azo under normoxia or hypoxia for 6 h (DAPI was used for nuclei staining). (B) Annexin V-FITC/Propidium Iodide (PI) dual-staining apoptosis analysis for HepG2 cells. The cells were incubated with varied concentrations of NR-azo under hypoxia. Viable cells are negative for both PI and annexin V (Q3-LL region); early apoptotic cells are PI negative and annexin V positive (in Q4-LR); late apoptotic/dead cells are positive for both PI and annexin V (in Q1-UR), while the damaged cells locate in region Q2-UL. (C) DNA (0.5 μg pBR322) crosslinking experiment upon treatment with different formulations. Concentration of SDT (if any): 100 μM. Concentration of NR-azo (if any): Lane 1 - 30 μM; Lane 2 - 0 μM; lane 3 - 10 μM; lane 4 - 15 μM; lane 5 - 20 μM; lane 6 - 30 μM; Concentration of CLB in lane 7: 30 μM.

Next, we investigated the hypoxia-triggered azo-cleavage in NR-azo and the subsequent generation of NR-NH_2_ in a cancer cell line (HepG2 cells) by fluorescent imaging. The cells were incubated in hypoxic atmosphere (in a hypoxic incubator chamber in which oxygen content was decreased to 0.1%) or in normoxia for 6 h, and then fluorescence images were acquired by using fluorescence microscope (Fig. 2A). Upon incubation with NR-azo under hypoxia, the HepG2 cancer cells displayed distinct intracellular red fluorescence; while under normoxia no red fluorescence could be observed in the cells (Fig. 2A). On the other hand, after the cells were incubated with the control chromophore (NR-CLB) under hypoxia condition, no intracellular red fluorescence could be observed, as shown in Fig. S16. This is because NR-CLB has no azo linkage, and hypoxia couldn't trigger the release of NR-NH_2_. These results indicate that, the intracellular red fluorescence is due to the generation of NR-NH_2_ in the cells under hypoxia. Moreover, cell viabilities under hypoxia or normoxia were determined by MTT assay, and the results are shown in Fig. S17. It is clear that under both hypoxia and normoxia, the free antitumor drug (nitrogen mustard) chlorambucil (CLB) exhibits the highest toxicity towards HepG2 cells due to the severe toxicity of CLB. Under hypoxia, NR-azo displayed dose-dependent toxicity against HepG2 cells, and the IC_50_ value of NR-azo was calculated as 40 μM; while NR-NH_2_ alone exhibited little toxicity against the cells. Whereas under normoxia, neither NR-azo nor NR-NH_2_ showed obvious cell toxicity. NR-azo's low cytotoxicity under normoxia and high cytotoxicity under hypoxia may be due to the fact that its azo linkage is cleaved under hypoxia, which consequently releases the active drug nitrogen mustard.

To evaluate the effect of NR-azo on the apoptosis of HepG2 cells under hypoxia, we used Annexin V-FITC/PI double-staining assay in flow cytometry analysis, and the results are displayed in Fig. 2B. Cell apoptosis (including the early apoptosis shown in Q4 and the late apoptosis shown in Q1 of each panel in Fig. 2B) was determined after the cells were incubated under hypoxia for 6 h. It can be seen that under hypoxia, NR-azo induces dose-dependent apoptosis; 5 μM of NR-azo leads to the apoptosis percentage of 11.7% and 30 μM of NR-azo results in the apoptosis of 40.1%. This result is in conformity with that of MTT assays and again supports the idea that the active drug nitrogen mustard can be released under hypoxia.

As a DNA interstrand cross-linking agent, nitrogen mustard can induce DNA damage, disturb cell cycles and cause strong cytotoxicity, and this is the source of cytotoxicity for the released drug. The interstrand DNA cross-linking capability of NR-azo was evaluated by agarose gel electrophoresis analysis using linearized plasmid DNA (pBR322). As shown in Fig. 2C, CLB, a commercially-available nitrogen mustard-based anti-cancer drug, displayed strong interstrand DNA cross-linking activity, and the resulting cross-linked pBR322 DNA is presented in the lower band (lane 7). The NR-azo or SDT alone, however, cannot lead to interstrand DNA cross linking (lane 1 and lane 2). In the presence of both NR-azo and SDT, the interstrand cross-linking ability for pBR322 displays a dosage-dependence enhancement (lane 3 to lane 6). These results again prove that the SDT-mediated cleavage of azo linker can cause the release of the nitrogen mustard drug which exhibits strong cytotoxic effect. Taken together, the above in-vitro results demonstrate that the NR-azo has the strong potential toward in vivo application for imaging hypoxia and therapy.

### Fluorescent and MSOT Imaging of hypoxia in vivo via intratumoral injection of molecular NR-azo

To test the capability of NR-azo for fluorescent and MSOT imaging of hypoxia in vivo, we respectively injected the molecular NR-azo or control into the tumor-bearing mice intratumorally and performed the fluorescent and MSOT imaging. The tumor-bearing mice models were established by subcutaneously injecting the mice with ca. 2 × 10^6^ HepG2 cells at the back for fluorescent and MSOT imaging.

**Figure 3:**
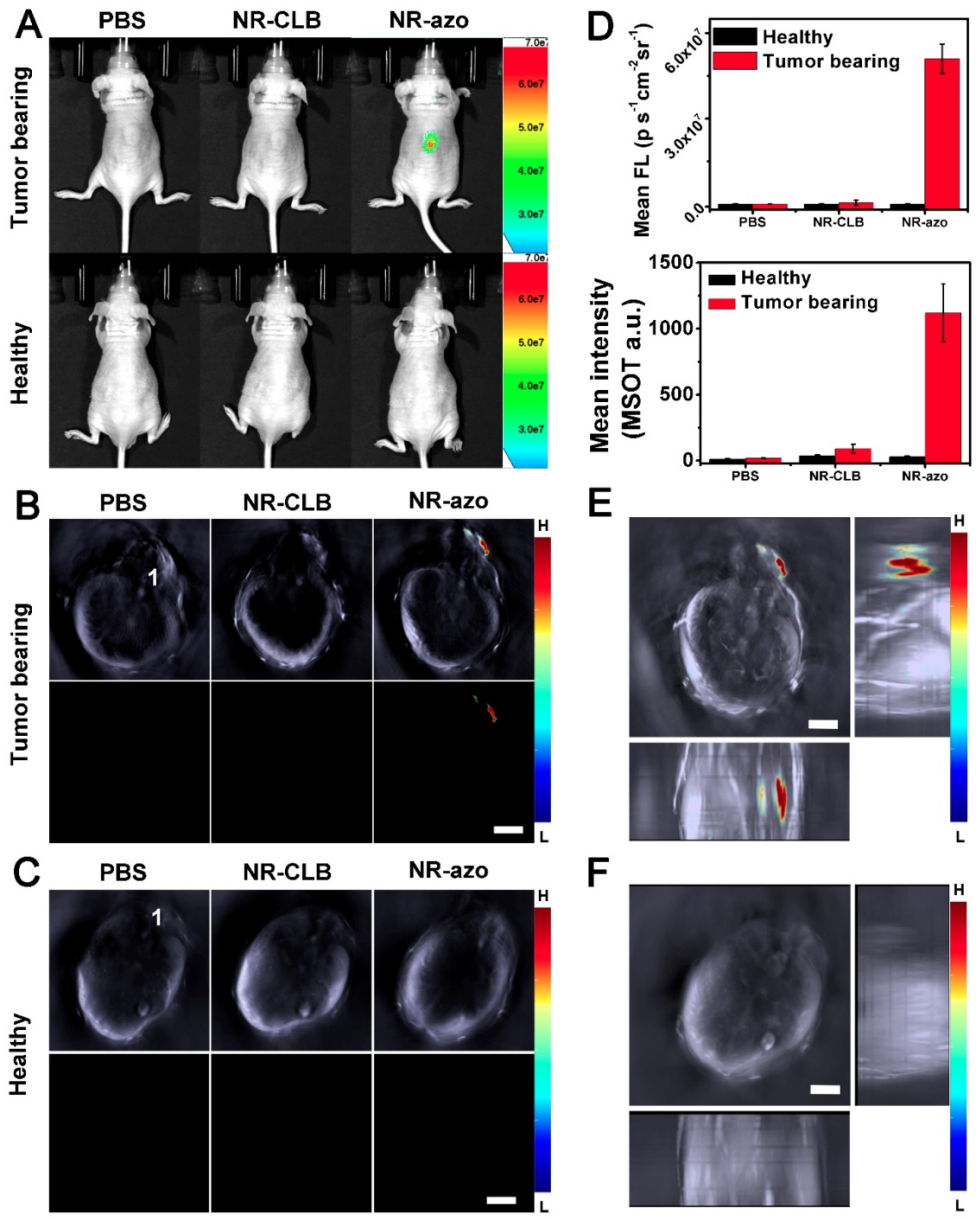
(A) Typical fluorescence images of tumor-bearing mice (upper row) and heathy mice (lower row) at 0.5 h upon intratumoral injection of PBS (200 μL), NR-CLB or NR-azo (8 mg/kg, in PBS with 1% DMSO). Excitation filter: 675 nm; Emission Filter: 710. (B) Cross-sectional MSOT images of the tumor-bearing mouse corresponding to those in the upper row of (a). 1: spinal cord. Scale bar = 5 mm. (C). Cross-sectional MSOT images of healthy mouse corresponding to those in the lower row of (A). 1: spinal cord. Scale bar = 5 mm. (D) Fluorescence (upper panel) and OA (lower panel) intensities upon injection of PBS, NR-CLB, NR-azo corresponding to those in (A) and (B). (E) Orthogonal-view 3D MSOT images of the tumor-bearing mouse injected with NR-azo corresponding to that in the upper row of (A). Scale bar = 5 mm. (F) Orthogonal-view 3D MSOT images of the healthy mouse injected with NR-azo corresponding to that in the lower row of (A). Scale bar = 5 mm.

As shown in Fig. 3A (upper row) and Fig. 3D, it is obvious that, when PBS or NR-CLB (the control chromophore without azo linkage) was injected intratumorally, no fluorescence could be observed; while after NR-azo injection, the fluorescence signal in the tumor region was quite significant, indicating NR-azo can respond to tumor hypoxia and correspondingly release NR-NH_2_. In contrast, as shown in the lower panel of Fig. 3A, for healthy mice (used as negative control), almost no fluorescence signal can be detected on the mice back where were injected with PBS, NR-CLB or even NR-azo, as there is no hypoxia in normal tissue on the mice back. In addition, the experiment with NR-NH_2_ as a control was performed as well, and the results are shown in Fig. S18; it can be seen that, at 0.5 h post injection, the NR-NH_2_ mainly remained in tumor site, which indicates the diffusion to other tissues is relatively slow.

Fig. 3B and 3C show the cross-sectional MSOT images of the mice the same as in Fig. 3A. The lower rows of Fig. 3B and 3C display the multispectrally resolved MSOT signal from NR-NH_2_ (the activated chromophore); and the upper rows display the overlay of MSOT signals from NR-NH_2_ (in color) with a single wavelength (800 nm) background image serving as the anatomical reference (in grayscale). For the healthy mice, upon injection with PBS, NR-CLB or NR-azo, almost no signal could be observed (Fig. 3C). While for the tumor-bearing mice injected with NR-azo, the multispectrally resolved signal of NR-NH_2_ was evidently strong (Fig. 3B). This is because tumor hypoxia triggers azo cleavage and the subsequent generation of NR-NH_2_, which correspondingly produces optoacoustic signals. One can also find that, the position of fluorescent signals all correspond well with that of MSOT signals (during MSOT imaging, the mice laid on their stomachs with slight tilt, the spine cord of each mouse was labeled with “1” to reflect the extent of tilt). Furthermore, we obtained the orthogonal-view 3D MSOT images, as shown in Fig. 3E and 3F. The optoacoustic signal from the tumor region of the mouse injected with NR-azo was much stronger compared to that in the healthy mouse. With the aid of the orthogonal-view 3D images, we could determine the volume and the relative position of solid tumor on the mice's back more directly and precisely. These results demonstrate that the chromophore NR-azo can respond to tumor hypoxia and can serve as a fluorescent/opotoacoustic dual-mode probe for tumor hypoxia in vivo. Moreover, to verify the biosafety of the chromophore, we performed histological analysis (H&E staining) of sections from main organs (heart, lung, liver, spleen and kidney) of healthy mice upon different treatments; Fig. S19 shows that there was no obvious difference between the mice group injected with PBS and the mice groups i.v. injected with NR-azo or NR-NH_2_. This confirms that both NR-azo and NR-NH_2_ are of high biosafety.

### Fluorescent and MSOT imaging of drug release from liposomal NR-azo and its action on tumor inhibition

**Figure 4:**
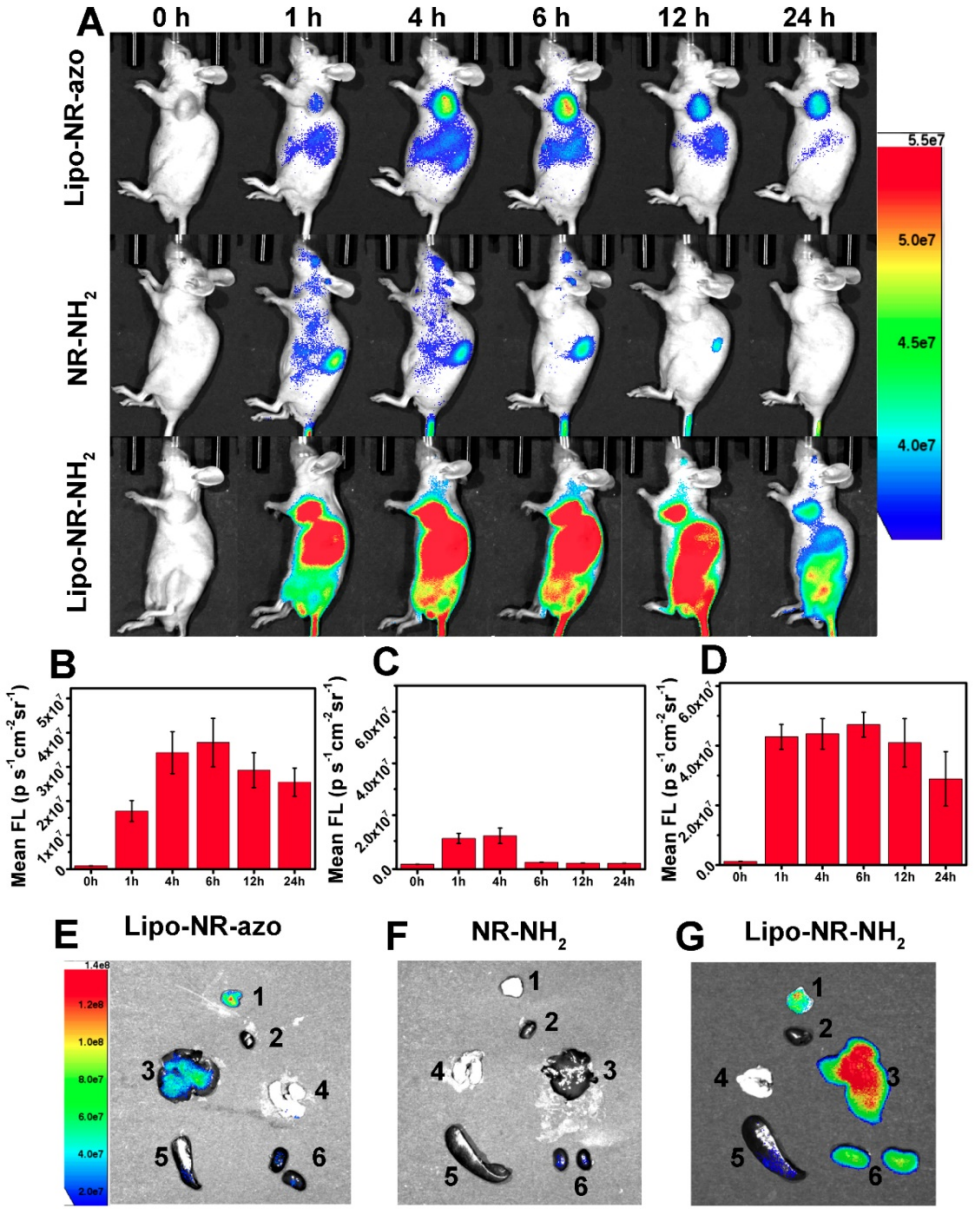
(A) Typical fluorescence images of HepG2 xenograft tumor-bearing mice upon intravenous injection of NR-NH_2_ (in PBS containing 1 % DMSO), Lipo-NR-azo (in PBS) and Lipo-NR-NH_2_ (in PBS) for different time. (B), (C) and (D) Mean fluorescence intensities at tumor site of the mice with intravenous injection of Lipo-NR-azo, NR-NH_2_ and Lipo-NR-NH_2_ respective. (E), (F) and (G) Ex vivo fluorescence images of major organs and tumor collected from the tumor-bearing mice sacrificed at 24 h after treatment with Lipo-NR-azo, NR-NH_2_ and Lipo-NR-NH_2_. (1: tumor, 2: heart, 3: liver, 4: lung, 5: spleen, 6: kidneys) (Exicitation filter: 675 nm).

Next, NR-azo molecules were encapsulated into liposomes (hereinafter referred to as Lipo-NR-azo) using phospholipids to ensure efficient accumulation of Lipo-NR-azo in tumor region (at left oxter) via the enhanced permeability and retention effect (EPR effect).^8^,^9^ The TEM images and particle size distribution determined by dynamic light scattering method are shown in Fig. S20. It is clear that, the size of the liposomal NR-azo is mainly in the range of 60~100 nm; and the size of nanoparticles plays an important role in EPR effect, 8,9 nanoparticles of this size would accumulate in tumor site via EPR effect. The Lipo-NR-azo nanoparticles are quite stable after being stored at 4 ºC for 14 day as confirmed through absorption spectra and size distribution measurement by DLS, as shown in Fig. S21. In addition, the stability of Lipo-NR-azo and NR-azo at 25 ºC and 37 ºC in PBS with or without serum has also been tested (Fig. S21), and it is clear they can remain stable for a few days under such conditions. And Lipo-NR-azo retained the optical properties of NR-azo (Fig. S22). Fig. 4A shows the fluorescence imaging of the tumor-bearing mice upon intravenous injection with Lipo-NR-azo, Lipo-NR-NH_2_ or molecular NR-NH2. For the mouse treated with Lipo-NR-azo, at 1 h post injection, fluorescent signal appeared in tumor region and liver area. Afterward, the fluorescence in tumor region become stronger and reached its maximum at 6 h post injection, and the fluorescent signal was still visible at 24 h post injection, which was due to the enhanced permeability and retention (EPR) effect. For the mouse treated with Lipo-NR-NH_2_, the fluorescence signal appeared in almost the whole body but mainly in the liver and the tumor site; at 24 h post injection, the fluorescence signal was still visible in the tumor site due to the EPR effect.

In contrast, for the mice treated with the molecular fluorophore NR-NH_2_ (Fig. 4A and Fig. S23), the fluorescence mainly resided in kidneys and was cleared out of the body more quickly. In order to further confirm the EPR effect of Lipo-NR-azo, the in vivo imaging and ex vivo imaging of major organs and tumors of the mice treated with NR-azo was performed as well, as shown in Fig. S24. For in vivo imaging of the mice treated with NR-azo, no obvious fluorescence was observed probably due to the quenching effect of azo bond and quick metabolic rate of small molecule. While ex vivo imaging of major organs and tumors showed that NR-azo was relatively quickly cleared out of body and could not accumulate efficiently in the tumor region. The fluorescence intensities at tumor site of the mice with intravenous injection of Lipo-NR-azo, NR-NH_2_ and Lipo-NR-NH_2_ respectively are shown in Fig. 4B-4D.

In addition, after intravenous injection of Lipo-NR-azo, molecular NR-NH_2_ or Lipo-NR-NH_2_ for 24 h the mice were sacrificed by CO_2_ exposure, the tumor and some organs were dissected for fluorescent imaging; and the ex vivo fluorescence imaging of main organs and tumor are shown in Fig. 4E-4G. For the mice injected with NR-NH_2_, weak fluorescent signal in kidneys could be observed; while for the mice injected with Lipo-NR-azo, strong fluorescent signal could be found in tumor site and in liver, and weak signal in kidneys. These results clearly indicate that, the liposomal NR-azo can accumulate in tumor region via EPR effect and generate fluorescence for monitoring the release of its active components.

**Figure 5:**
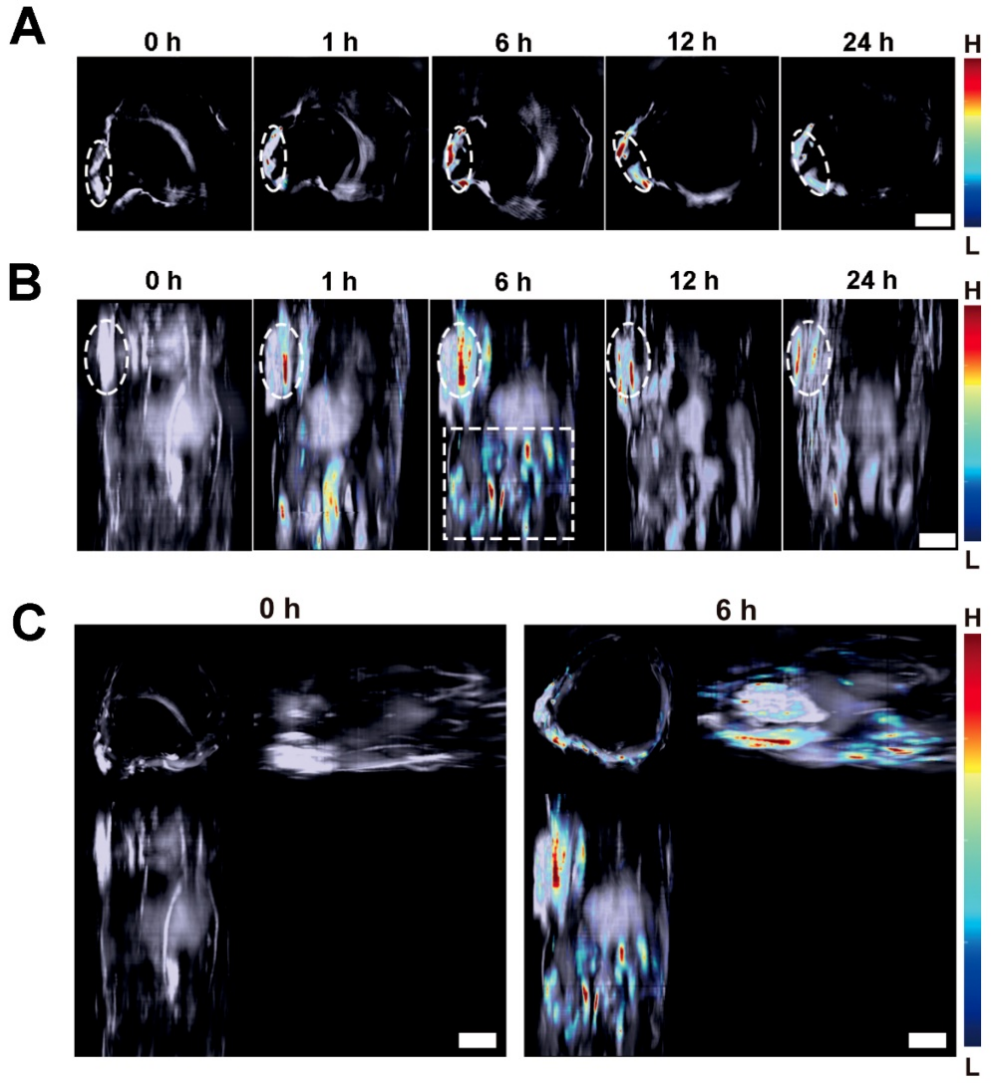
(A) Typical cross-sectional MSOT images at tumor region of a tumor-bearing mouse at varied time upon intravenous injection of Lipo-NR-azo (in PBS). The tumor region was marked with white dotted circle. (B) Typical vertical view of a part of trunk of the mouse at varied time upon injection of Lipo-NR-azo. The white dotted-line rectangle represents the upper abdominal region of the mouse. (C) A typical 3D MSOT imaging of a part of trunk of the tumor-bearing mouse at 0 h and 6 h post injection of Lipo-NR-azo (in PBS). Scale bar = 5 mm.

On the other hand, MSOT imaging was performed to observe the response of the Lipo-NR-azo to hypoxia in tumor tissue. Fig. 5A shows the cross-sectional MSOT images at tumor region at varied time post intravenous injection of Lipo-NR-azo. There is almost no MSOT signal of the activated fluorophore (NR-NH_2_) at 0 h. The MSOT signal began to emerge at 1 h at tumor region, and then gradually intensifies at longer time until it reached its maximum at 6 h. The MSOT technique allows us to use computed tomography scans to gain 3D information of the biodistribution of the activated chromophore. In this study, we obtained a stack of cross-sectional MSOT images by scanning a part of trunk (from oxter to the upper abdominal region) of a tumor-bearing mouse upon intravenous injection of Lipo-NR-azo and rendered the cross-sectional (tomographic) information into maximum intensity projection (MIP) images. Fig. 5B and 5C show the vertical view and three-view images at varied time upon intravenous injection of Lipo-NR-azo, respectively. As can be seen in Fig. 5B (and Fig. S25), the MSOT signal at tumor site emerged at 1 h and reached its maximum at 6 h and remained relatively strong at 24 h. In addition to the tumor region, the MSOT signal could also be observed in liver region (upper abdominal region), but it decreased quickly thereafter and at 12 h the signal became very weak. Moreover, as we can see in Fig. 5C, the MIP MSOT images for the trunk of a tumor-bearing mouse can provide 3D information for the tumor hypoxia. Compared to the fluorescent imaging, the MSOT allows us to assess the size and location of the hypoxia more precisely due to the higher spatial resolution in depth as well as the less scattering nature of the ultrasound. It is well known that the solid tumors are heterogeneous masses with both normal cells and neoplastic cells, and the hypoxia is also of heterogeneity; the MSOT images, especially the 3D images, can reflect the heterogeneity of the hypoxia in tumor tissue.

**Figure 6:**
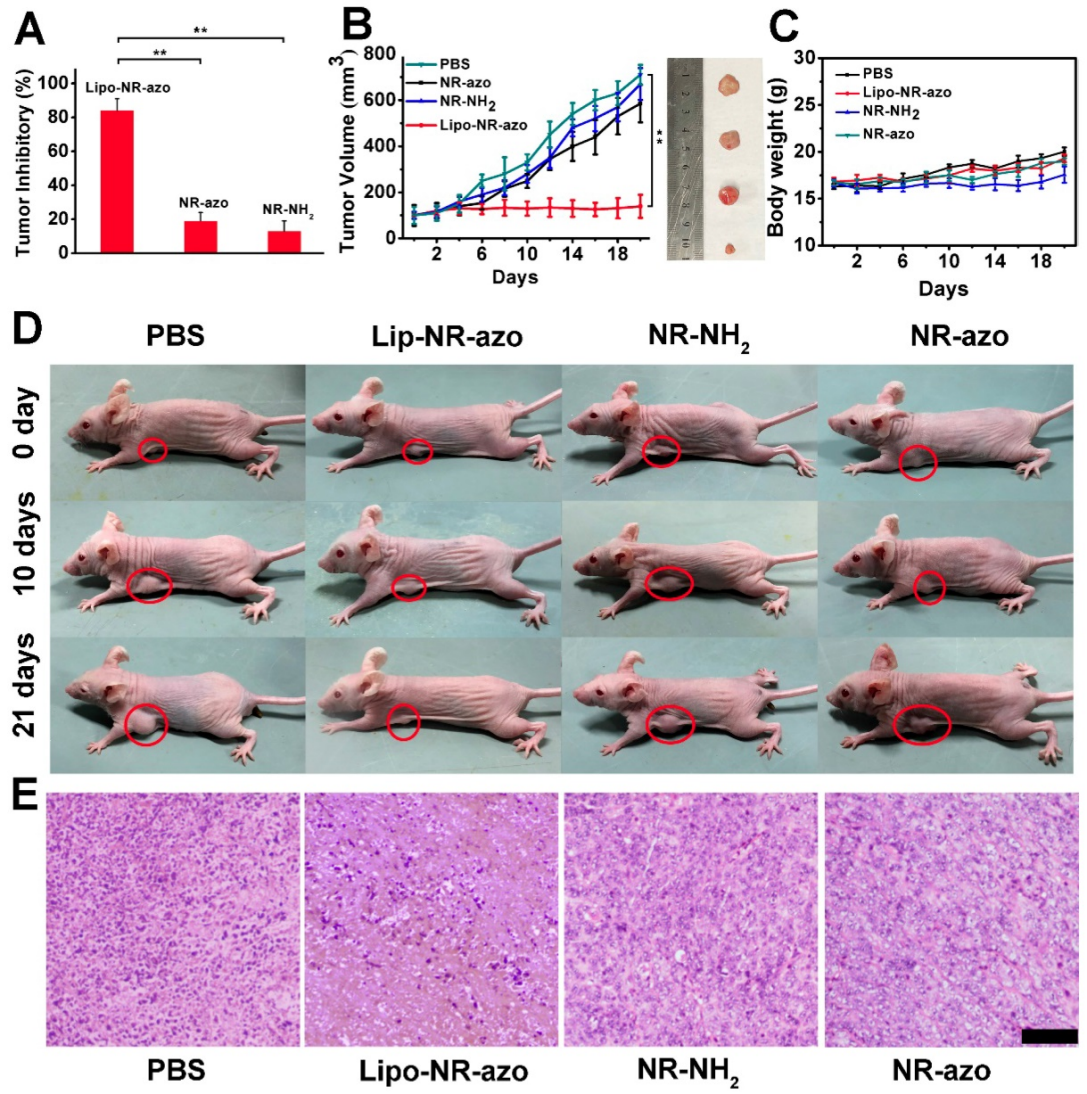
(A) Tumor inhibition rate of each group. (n = 6 per group). (B) Tumor volume of tumor-bearing mice upon intravenous injection of PBS, Lipo-NR-azo, NR-NH_2_ or NR-azo. Data represent mean ± SD from six independent experiments. For each group, the photograph of a typical dissected tumor was given in the right images. (C) Body weight of the mice in various groups during treatment. (D) photographs of live mice treated in different formulations during the 21 day's period. (E) H&E staining analysis of tumor tissue sections for different groups of the mice. Scale bar = 100 μm. Columns represent mean ± SD. The *P*-values (*P < 0.05, **P < 0.01) were determined using two-sided Student's *t*-test.

In order to evaluate the tumor inhibition efficacy of the liposomal system, 24 tumor-bearing mice were randomly assigned into four groups. PBS, lipo-NR-azo (16.1 mg/kg, which is equivalent to 8 mg/kg NR-azo), NR-azo (8 mg/kg) or NR-NH_2_ (8 mg/kg) were i.v. injected into the mice every other day. During the 21-day therapy course, the body weights for all groups were recorded. After the therapy, the mice were humanely sacrificed by CO_2_ exposure and the tumors and some major organs were dissected, weighted and photographed. For the mice treated with Lipo-NR-azo, the tumor growth was significantly suppressed compared to other groups, and the tumor inhibitory rate (TIR) for Lip-NR-Azo group (82.2%) was much higher than that for other groups (including the NR-azo group with its TIR at 18.7%), as shown in Fig. 6A and 6B. It had also been found that, during the treatment period all the groups show slight weight gain (Fig. 6C). The visualized tumor inhibition efficacies for the groups are shown in Fig. 6D. In comparison, the treatment with Lipo-NR-CLB was conducted and the result is shown in Fig. S26; it can be seen that, for Lipo-NR-CLB treatment, no obvious treatment effect could be observed compared to Lipo-NR-azo, since no active drug could be released. The histological analysis results (H&E staining) for the tumor tissue sections are shown in Fig. 6E. For the group treated with Lipo-NR-azo, the number of tumor cells within the microscopic field is much less than other groups and obvious necrosis can be observed. These results indicate that only the treatment with Lipo-NR-azo can result in significant tumor inhibition.

## Conclusions

In summary, we have developed an activatable chromophore NR-azo and then prepared the liposome encapsulated with NR-azo that can specifically respond to tumor hypoxia, and thereby releases the fluorophore NR-NH_2_ and the active anti-cancer drug. The activated fluorophore can be employed for detecting and imaging tumor hypoxia both fluorescently and optoacoustically, while the released drug can achieve tumor inhibition in tumor-bearing mice model. This work could offer an optoacoustic and fluorescent system for not only imaging tumor hypoxia but also achieving tumor inhibition. Hence the approach herein could offer useful insights for designing chromophores for detecting other biomarkers and therapy.

## Supplementary Material

Supplementary figures.
