# Supplementary material for: An Activatable Near-Infrared Chromophore for Multispectral Optoacoustic Imaging of Tumor Hypoxia and for Tumor Inhibition
Source: Theranostics. 2019 Sep 25;9(24):7313–24. doi: 10.7150/thno.36755 (PMC6831286; doi:10.7150/thno.36755)
Supplement: Supplementary file 1 — Supplementary figures. [file thnov09p7313s1.pdf]

Electronic Supplementary Information

An Activatable Near-Infrared Chromophore for Multispectral Optoacoustic Imaging of Tumor Hypoxia and for Tumor Inhibition

Jing Huang, Yinglong Wu, Fang Zeng*, Shuizhu Wu*

State Key Laboratory of Luminescent Materials & Devices, College of Materials Science & Engineering, South China University of Technology, Guangzhou 510640, China. E-mail: shzhwu@scut.edu.cn; mcfzeng@scut.edu.cn

Table of Contents

Experimental section S3
Scheme S1. Synthetic route S9
Figure S1. ^{1}H NMR spectrum of compound 2 .... S10
Figure S2. ^{13}C NMR spectrum of compound 2 .... S10
Figure S3. Mass spectrum of compound 2 .... S11
Figure S4. ^{1}H NMR spectrum of compound 3 .... S11
Figure S5. ^{13}C NMR spectrum of compound 3 .... S12
Figure S6. Mass spectrum of compound 3 .... S12
Figure S7. ^{1}H NMR spectrum of compound 4 .... S13
Figure S8. ^{13}C NMR spectrum of compound 4 .... S13
Figure S9. Mass spectrum of compound 4 .... S14
Figure S10. ^{1}H NMR spectrum of control chromophore S15
Figure S11. Mass spectrum of control chromophore S15
Figure S12. Absorption and fluorescence spectra S16
Figure S13. Fluorescence spectra S16
Figure S14. Absorption and fluorescence spectra S17
Figure S15. Selectivity experiment S17
Figure S16. Fluorescence images for HepG2 cells S18
Figure S17. MTT experiment S18
Figure S18. In vivo fluorescence imaging S19

Figure S19. Histochemical and immunohistochemical analysis.......................... S20
Figure S20. Particle size distribution and TEM images....................................... S21
Figure S21. Stability of Lipo-NR-azo and NR-azo... S22
Figure S22. Spectral properties... S23
Figure S23. In vivo fluorescence imaging S23
Figure S24. Fluorescent images S24
Figure S25. Mean MSOT intensities.. S24
Figure S26. Tumor inhibition experiment... S25
References.. S26

Experimental section

Synthetic procedures

Compound 1 and compound 2:

Compound 1 was prepared according to established protocols.[1] Briefly, to the solution of Ac_2O, the mixture of 2-chloro-1-formyl-3-(hydroxymethylene)cyclohex-1-ene (344 mg, 2 mmol), 1,2,3,3-tetramethyl-3H-indolium iodide (1.2 g, 4 mmol) and NaOAc (324 mg, 4 mmol) were added and heated to 90 ^{o}C for 1 h, the precipitate was washed by distilled water and diethyl ether. The product was used for next step without further purification. The yield was 90 %.

In a flask, 3-nitrophenol (2.2 g, 16.4 mmol) and Et_3N (0.7 mg, 7 mmol) was dissolved in 10 mL DCM, the mixture was stirred at room temperature under nitrogen atmosphere for 30 min. After that, compound 1 in 10 mL DCM was introduced to the mixture via a syringe, then the reaction mixture was stirred at room temperature for 24 h. After reaction completed the solvent was evaporated under reduced pressure, and the precipitate was dispersed in 20 mL MeOH, for further used in the next step.

$SnCl_2$ (3 g, 17 mmol) was dissolved in 3 mL concentrated HCl, then added to the above solution under nitrogen atmosphere. The solution was heated to reflux and stirred overnight. When the reaction was completed, the solvent was evaporated under reduced pressure; the precipitate was dissolved by DCM and washed with saturated $NaHCO_3$ solution for three times and dried over Na_2SO_4. The solvent was removed, and the residue was purified by column chromatography with DCM/MeOH (100:1 to 10:1) as eluent, affording compound 2 as dark green solid (214 mg, yield 66 %). ^{1}H NMR (600 MHz, CD_3OD) δ 8.61 (d, *J* = 14.3 Hz, 1H), 7.55 (d, *J* = 7.2 Hz, 1H), 7.51 (s, 1H), 7.47 – 7.43 (m, 1H), 7.38 – 7.30 (m, 3H), 6.76 (dd, *J* = 8.5, 2.0 Hz, 1H), 6.71 (d, *J* = 1.6 Hz, 1H), 6.22 (d, *J* = 14.3 Hz, 1H), 3.69 (s, 3H), 2.78 – 2.75 (m, 2H), 2.70 (t, *J* = 6.0 Hz, 2H), 1.95 – 1.90 (m, 2H), 1.78 (s, 6H). ^{13}C NMR (151 MHz, CD_3OD) δ 174.75, 163.33, 156.17, 154.88, 142.80, 142.67, 141.01, 138.26, 129.53, 128.52, 125.33, 123.19, 121.97, 114.52, 114.44, 113.69, 110.83, 99.82, 97.68, 53.40, 49.34, 30.44, 28.26, 27.25, 23.81, 20.53 ppm. MS (ESI): $C_{26}H_{27}N_2O^+$ [M^+], *m/z* Found: 383.0.

Compound 3:

Compound 2 (200 mg, 0.39 mmol) was dissolved in 30 mL (DCM:MeCN:H_2O=5:1:0.01), to this stirred solution, $NaNO_2$ (55 mg, 0.8 mmol) was added, the mixture was stirred for 15 min at 0 ^{o}C followed by adding amidosulfonic acid (67 mg, 0.7 mmol) and stirred for another 20 min. Subsequently, *N*-phenyldiethanolamine (705 mg, 3.9 mmol) in 10 mL MeCN was added and the solution was stirred for 4 h at 0 ^{o}C. After reaction was completed, the solvent was removed under reduced pressure, the residue was purified by column chromatography with DCM/MeOH (50:1 to 10:1) as eluent, affording compound 3 as black solid (96 mg, yield 43 %). ^{1}H NMR (500 MHz, CD_3OD) δ 8.20 (d, *J* = 15.1 Hz, 1H), 7.69 – 7.63 (m, 2H), 7.48 (d, *J* = 8.1 Hz, 1H), 7.43 – 7.33 (m, 4H), 7.20 (d, *J* = 0.8 Hz, 1H), 7.13 (s, 1H), 7.05 (d, *J* = 7.8 Hz, 1H), 6.54 (d, *J* = 9.1 Hz, 2H), 5.82 (d, *J* = 15.1 Hz,

1H), 3.82 (t, J = 5.9 Hz, 4H), 3.70 (s, 3H), 3.67 (t, J = 5.9 Hz, 4H), 2.59 – 2.49 (m, 2H), 2.07 (d, J = 5.6 Hz, 2H), 1.79 (s, 6H), 1.72 – 1.68 (m, 2H). ^{13}C NMR (151 MHz, CD_3OD) δ 178.07, 159.08, 154.27, 153.12, 152.06, 144.90, 142.73, 141.80, 141.72, 131.04, 130.43, 128.85, 128.15, 127.54, 122.77, 122.22, 114.71, 111.80, 111.68, 105.88, 105.47, 64.75, 59.00, 53.83, 53.49, 50.63, 50.37, 31.50, 28.82, 26.85, 23.06, 19.65. ppm. MS(ESI): $C_{36}H_{39}N_4O_3^+$ [M^+], *m/z*, Found: 575.2.

Compound 4:
Compound 3 (115 mg, 0.2 mmol) was dissolved in 5 mL DCM, the $SOCl_2$ (118 mg, 2 mmol) was added at 0 ^oC and stirred for 10 min, then the reaction mixture was heated to reflux for 3 h. After that, the solution was cooled to room temperature, and 30 mL MeOH was added to quench reaction, then the solvent was evaporated and the product was isolated by column chromatography with DCM/MeOH (100:1) as eluent, affording compound 4 as black solid (105 mg, yield 86 %). ^{1}H NMR (600 MHz, $CDCl_3$) δ 8.58 (d, J = 15.0 Hz, 1H), 7.90 (d, J = 8.7 Hz, 2H), 7.74 (dd, J = 8.2, 1.1 Hz, 1H), 7.59 (s, 1H), 7.52 – 7.46 (m, 3H), 7.44 (d, J = 8.2 Hz, 1H), 7.40 (t, J = 7.3 Hz, 1H), 7.11 (s, 1H), 6.79 (d, J = 8.9 Hz, 2H), 6.70 (d, J = 15.1 Hz, 1H), 4.02 (s, 3H), 3.89 (t, J = 6.8 Hz, 4H), 3.73 (t, J = 6.8 Hz, 4H), 2.80 – 2.66 (m, 4H), 1.95 – 1.87 (m, 2H), 1.80 (s, 6H). ^{13}C NMR (151 MHz, $CDCl_3$) δ 178.63, 159.93, 154.61, 153.36, 149.65, 146.14, 144.68, 142.17, 141.95, 131.35, 131.14, 129.44, 127.93, 127.87, 123.10, 122.18, 120.24, 116.30, 113.57, 111.93, 108.67, 107.03, 53.54, 50.86, 40.34, 34.12, 31.94, 29.70, 29.36, 28.07, 24.40, 22.69, 20.28, 14.10 ppm. MS(ESI): $C_{36}H_{37}Cl_2N_4O^+$ [M^+], *m/z*, Found: 611.2

Control chromophore
Chlorambucil (40 mg, 0.13 mmol) and DIPEA (50 mg, 0.39 mmol) was dissolved in 5 mL anhydrous DCM, the mixture was allowed to stir at ambient temprature for 30 min, then compound 2 (50 mg, 0.13 mmol) was dissolved in 5 mL anhydrous DCM was introduced to the solution, then the reaction proceeded for another 3h, after the reaction completed, solvent was evaporated and the residue was suffer from column chromatography with DCM/MeOH (200:1) as eluent, affording control fluorophore as blue solid (74 mg, yield 86 %). ^{1}H NMR (600 MHz, $CDCl_3$) δ 8.69 (d, J = 14.6 Hz, 1H), 8.56 (s, 1H), 8.03 (d, J = 7.5 Hz, 1H), 7.60 (s, 1H), 7.47 (d, J = 7.4 Hz, 1H), 7.42 (t, J = 7.6 Hz, 1H), 7.33 (t, J = 7.4 Hz, 1H), 7.26 – 7.23 (m, 2H), 7.15 – 7.10 (m, 3H), 7.07 (d, J = 8.5 Hz, 2H), 6.20 (d, J = 14.6 Hz, 1H), 3.76 (s, 3H), 3.72 – 3.65 (m, 4H), 3.64 – 3.58 (m, 4H), 2.71 – 2.68 (m, 2H), 2.62 (dd, J = 9.5, 5.4 Hz, 4H), 2.57 (d, J = 7.6 Hz, 2H), 2.52 (t, J = 7.4 Hz, 2H), 2.36 (t, J = 7.4 Hz, 2H), 1.77 (s, 6H) MS(ESI): $C_{40}H_{44}Cl_2N_3O_2^+$ [M^+], *m/z*, Found: 669.0

Instrumentation

^{1}H and ^{13}C NMR spectra were recorded with a Bruker Avance 600 MHz nuclear magnetic resonance spectrometer. Mass spectra were measured with a Bruker LCQ DECA XP high performance liquid chromatography-mass spectrometer using the

solvents of the chromatographic grade methanol and deionized water. Fluorescence spectra were recorded on a Hitachi F4600 fluorescence spectrometer. The quartz cells were selected to have a length and width of 1.0 cm. Absorption spectra and fluorescence spectra were obtained on Hitachi U-3010 UV-Vis spectrophotometer and Hitachi F-4600 Fluorescence Spectro-photometer. The quartz cells were selected to have a length and width of 1.0 cm. Fluorescence images were obtained using an Olympus IX 71 with a DP72 color CCD. HPLC analysis was performed on Agilent Technologies 1260 Infinity High Performance Liquid Chromatography. Flow cytometry were performed on a BD CS6 flow cytometer. Particle size/distribution was determined with Malvern Nano-ZS90 particle size analyzer via dynamic light scattering (DLS) mechanism. Transmission electronic microscopy experiment (TEM) was conducted on a JEM-2010HR transmission electron microscopy. Agarose gel electrophoresis was visualized and photographed on Bio-Rad Gel Dox XR+ Imaging System. Fluorescent whole-body imaging was performed on an AMI small animal fluorescence imaging system (Spectral Instruments Imaging Co.) with an excitation filter of 675 nm and an emission filter of 710 nm. All in-vitro phantom and in vivo mouse optoacoustic imaging experiments were performed on a multispectral optoacoustic tomographic imaging system (inVision 128, iThera Medical GmbH).

Spectroscopic methods

The chromophore was dissolved in DMSO to prepare stock solution (1 mM). Absorption and fluorescence spectra were examined in phosphate-buffered saline (10 mM PBS, pH=7.4) containing 1% DMSO as co-solvent upon excitation at 680 nm. The *in vitro* hypoxic condition (measurements involving MLM and STD in solutions) was prepared by bubbling nitrogen gas into the solutions for 30 min.[2] Mouse liver microsomes (MLM) was prepared according to published literature.[3,4] For the in vitro MLM reduction experiment, MLM (50 μg/mL) was pre-incubated at 37 ^{o}C for 5 min, then 10 μM NR-azo and co-factor 100 μM NADPH was added, then the time-dependent fluorescence spectra were recorded under hypoxia condition.

Preparation of Lipo-NR-azo

First, NR-azo (10 mg) was dissolved in 100 μL DMSO, then 1.5 mL THF was added. After that, DSPE-PEG 2000 (10 mg) was added into the solution, then the solution was cooled to 4 ^{o}C, under sonication, and the mixture was added dropwise into 10mL water and stirred for 24 h at room temperature. After that THF was removed under reduced pressure and then the resultant solution was dialyzed against water at room temperature for 24 h. The nanoparticle suspension was centrifuged at 12000 rpm for 15 min at 4 ^{o}C and the liposomal particles of Lipo-NR-azo were collected. Afterwards, the particles were washed by water for three times, and then resuspended in PBS (10 mL) for further use. The loading amount of NR-azo was determined based on the UV absorbance at 575 nm. The drug loading efficiency (DLE) is calculated as followed:

DLE = (mass of loaded NR-azo)/(mass of nanoparticles) × 100 %

The determined DLE for lipo-NR-azo was 49.7 %.

Linearization of plasmid pBR322 by EcoR I

At a humidified incubator (37 ^oC, 5% CO_2), supercoiled pBR322 DNA (30 μL, 30 μg) was incubated with EcoR I and EcoR I buffer (10X, 20 μL). After incubation for 3 h, NaOAc (40 μL, 30 M) and ethanol 1400 μL were added, then the solution was incubated at -20 ^oC overnight. Afterwards the solution was centrifuged at 12000 rpm for 15 min at 4 ℃, and then the supernatant was removed. After that, the residue was lyophilized to obtain the white solid. The finally obtained linearized DNA was suspended in 100 μL sterile H_2O.

Agarose gel electrophoresis

To the mixture of 1.2 g agarose, 0.015 mL solargel red and 150 mL tris-boric acid-EDTA buffer (50 mM) were added. The agarose solution was heated by microwave for 5 minutes to the extent that the agarose was dissolved completely, and then the solution was poured into the sizing groove and cooled down for 0.5 h at room temperature. The solidified gel was soaked into tris-boric acid-EDTA buffer (50 mM).

The DNA samples (1 μg/sample) were pre-treated with NR-azo, SDT, CLB, or NR-azo/SDT respectively in 10μL PBS (10 mM PBS, pH=7.4) at 37 ^oC, and the reaction was allowed to proceed for 2 h, then the solution was diluted to final volume of 20 μL for agarose gel electrophoresis.

The DNA samples with loading buffer (0.25 % bromophenol blue: glycerinum/tris-boric acid-EDTA buffer = 1:1) were added into the wells and run for 1 h at a voltage value of 100 V, then the gel was washed with water and visualized by the Gel-DocTM XR+ Image System.

Cell experiment

Cell culture HepG2 cells were cultured in Dulbecco's modified eagle medium (DMEM) supplemented with 10% FBS (fetal bovine serum,), 1% penicillin and streptomycin at 37 °C in a humidified atmosphere containing 5 % of CO_2, with medium changed every 2 days. Cells were passaged at 80 % ~ 90 % confluency using a 0.25 % trypsin/0.20 % EDTA solution. As for cell culture under hypoxia (O_2 concentration of 0.1%) was generated with an Anaero Pack® (Mitsubishi Gas Chemical Company, Inc.) and a 2.5 L hypoxic incubator chamber (Mitsubishi Gas Chemical Company, Inc.).

Cell viability assay Cell viabilities of free chlorambucil, NR-azo and NR-NH_2 toward HepG2 cells were evaluated by MTT assays. Briefly, HepG2 cells were seeded in 96-well plate (5000 cells/well), the cells were incubated with varied concentration of different formulations. After incubation, the plates were washed with PBS three times then treated with 0.5mg/mL MTT (in DMEM) for 4 h, the resulting formazan crystals were dissolved in DMSO and then

measured the absorption at 570 nm.

Cell imaging HepG2 cells were seeded in 6-well plate in DMEM supplemented with 10 % FBS for 24-h incubation. Cells on the plate were incubated to 70 % ~ 80 % confluence. NR-azo was then added and pre-incubated for 3 h. The 6-well plates were washed with PBS for three times to remove redundant probe, Then the cells were incubated in hypoxic/normoxic atmosphere for 6 h, and then fluorescence images were acquired by using fluorescence microscope.

Apoptosis analysis by annexin V-FITC and Propidium Iodide (PI) double staining HepG2 cells were seeded in 6-well plate at a density of 1 × 10^6 cells/well for 12 h at 37 °C. When the cell density reached 80~90% of confluence, they were treated with NR-azo (0-50 μM in medium) under hypoxia condition for 6 h and then the cells were washed with PBS once and then trypsinization before being harvested via centrifugation at 2000 rpm for 10 min. Subsequently, 1 mL PBS was further added and then mixed with Annexin V Binding buffer (400 μL) containing 5 μL Annexin V-FITC and 10 μL PI. The flow cytometry was performed using BD CS6 flow cytometer and the data were analysed using the BD Biosciences software.

Phantom optoacoustic imaging

Phantom optoacoustic imaging was performed on MSOT system (inVision 128 from iThera Medical GmbH). Briefly, the test solutions containing 10 μM NR-azo, MLM (50 μg/mL) and NADPH (100 μM) were stirred under nitrogen atmosphere for different time at 37 °C, and then added into NMR sample tubes for phantom optoacoustic imaging.

Animal experiment

All animal experiments were carried out in Laboratory Animal Centre of South China Agricultural University, and the experiment protocols have been approved by the Animal Ethics Committee of South China Agricultural University in conformity with the Guidelines for Care and Use of Laboratory Animals of Guangdong Province. BALB/c nude mice (5- 6 weeks old) were supplied by Guangdong Medical Laboratory Animal Center (GDMLAC). Animals were ensured to receive standard care (including aseptic environment, light/dark cycle of 12 h, autoclaved rodent diet and sterile water and) in Laboratory Animal Center of South China Agricultural University. Mice were randomly chosen to establish animal models for experimental investigation. Mice were randomly divided into groups with 6 mice per group. For lethal experimental procedures, mice were euthanized upon being exposed to carbon dioxide gas.

Mouse model The nude mice received subcutaneous injection of HepG2 cells (100 μL, 2 × 10^6 per mouse) on the left oxter or on the back. The tumor

inhibition experiment began when the tumor volume reached 100 mm^3. The tumor size was calculated by following equation: $V(mm^3) = L(mm) \times W^2/2$ (L: length, W: longest width, V: tumor volume), and the tumor inhibitory rate (TIR) was calculated by TIR (%) = (mean tumor weight of control group - mean tumor weight of experimental group)/mean tumor weight of control group.

In vivo and ex vivo fluorescence imaging

Fluorescence imaging was conducted using Ami small animal imaging system supplied by Spectral Instruments Imaging Co.. As for *in vivo* fluorescence imaging of the mice with subcutaneous injection, the chromophore (8 mg chromophore/kg body weight, in PBS containing 1% DMSO) was administered through subcutaneous intratumoral injection, and imaging was performed at 0.5 h post injection. As for *in vivo* fluorescence imaging of the mice with i.v. injection through tail vein, the dosage of Lipo-NR-azo is 16.1 mg/kg (which is equivalent to 8 mg/kg NR-azo), and the dosage of NR-NH_2 is 8 mg/kg, and fluorescence imaging was taken 1 h, 4 h, 6 h, 12 h and 24 h post injection. The mice were sacrificed by CO_2 exposure, then tumors and other organs were dissected and photographed by fluorescence imaging system.

MSOT imaging

Animal optoacoustic imaging was carried out by using MSOT system (inVision 128 from iThera Medical GmbH). Mice were anesthetized with continuous oxygen and 1% isoflurane via mouse breathing mask and put in the prone position in a 34 °C water bath. In one experiment, for the tumor-bearing mice model, the chromophore (8 mg/kg body weight, in PBS containing 1% DMSO) was administered through subcutaneous intratumoral injection; the mice were anesthetized and placed in the prone position in animal holder for imaging at 30 min post injection. Cross-sectional images were acquired through the whole tumor with a step size of 0.2 mm. Imaging wavelengths selected conforming to the major turning points in NR-NH_2's and hemoglobin's absorption spectra included 680 nm, 690 nm, 700 nm, 710 nm, 730 nm, 760 nm, 800 nm (background) and 850 nm. We recorded 5 frames at every imaging wavelength. In another experiment, for the tumor-bearing mice model, the Lipo-NR-azo (16.1 mg/kg) was administered via intravenous injection through tail vein; the mice were anesthetized and placed in the prone position in animal holder for imaging at 30 min post injection. Cross-sectional images were acquired through the whole tumor with a step size of 0.2 mm. Imaging wavelengths selected conforming to the major turning points in NR-NH_2's and hemoglobin's absorption spectra included 680 nm, 690 nm, 700 nm, 710 nm, 730 nm, 760 nm, 800 nm (background) and 850 nm. We recorded 5 frames at every imaging wavelength. After the MSOT images were generated, the z-stack was rendered as orthogonal-view three-dimensional images (guided ICA spectral unmixing was utilized to separate signals coming from the generated fluorophore and those from the photo-absorbing tissue elements in the body (e.g. hemoglobin)).

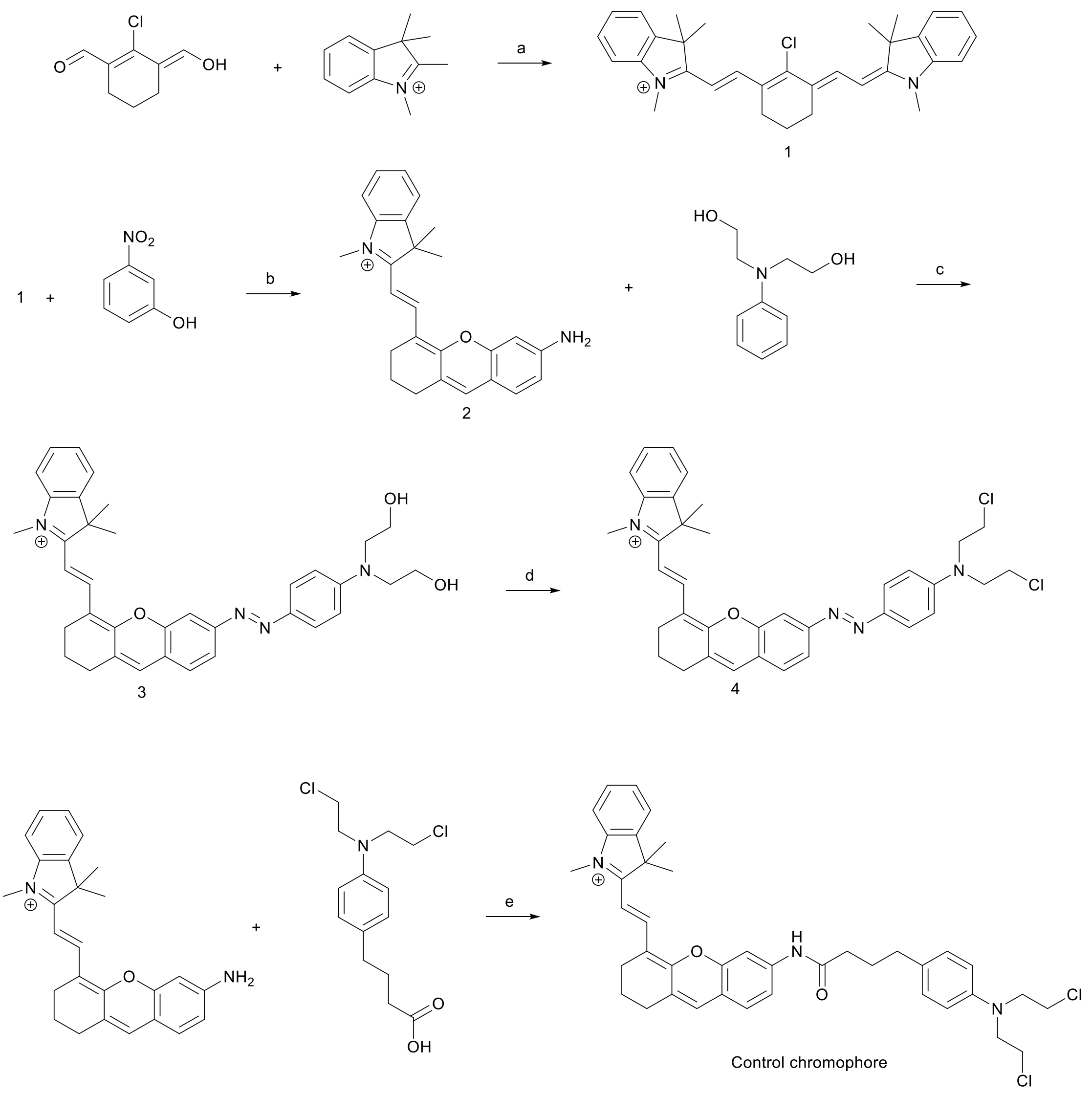

Scheme S1. Synthetic routes of compound 4 (NR-azo) and the control chromophore (NR-CLB). Reagents and conditions: a) NaOAc, Ac_2O, 90 °C, 1 h; b) Et_3N, DCM, r.t, 24 h; $SnCl_2$, HCl, MeOH, reflux, overnight; c) $NaNO_2$, amidosulfonic acid, MeCN/DCM, 0 °C, 2 h; d) $SOCl_2$, DCM, 45 °C, 2 h, e) HATU, DIPEA, r.t. 3 h.

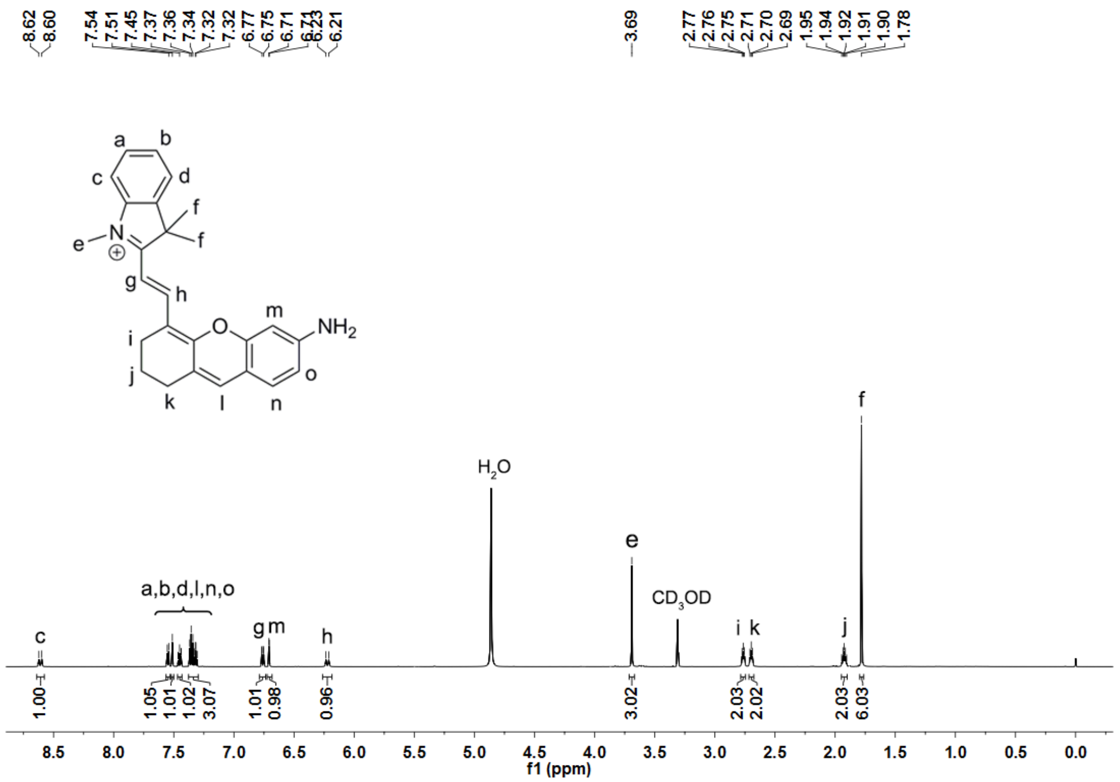

Figure S1. ^{1}H NMR spectrum of compound 2 (in CD_3OD).

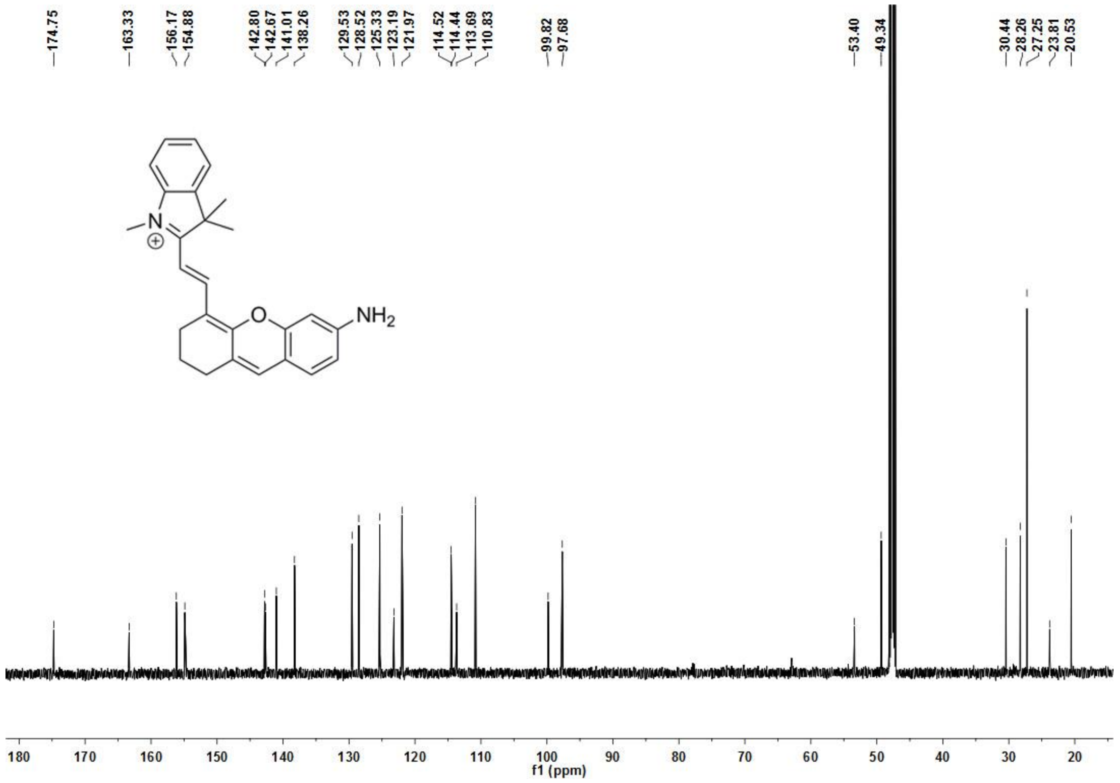

Figure S2. ^{13}C NMR spectrum of compound 2 (in CD_3OD).

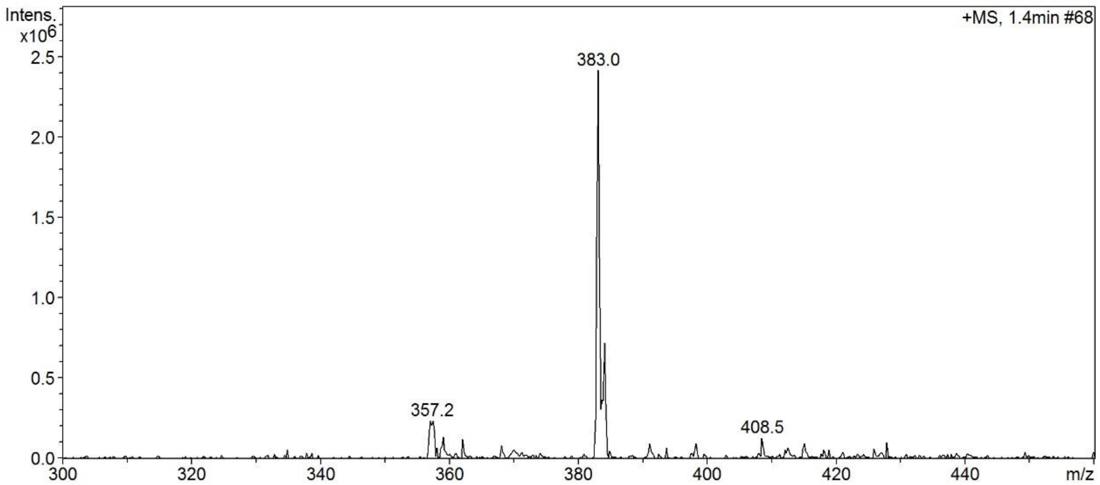

Figure S3. MS spectrum of compound 2.

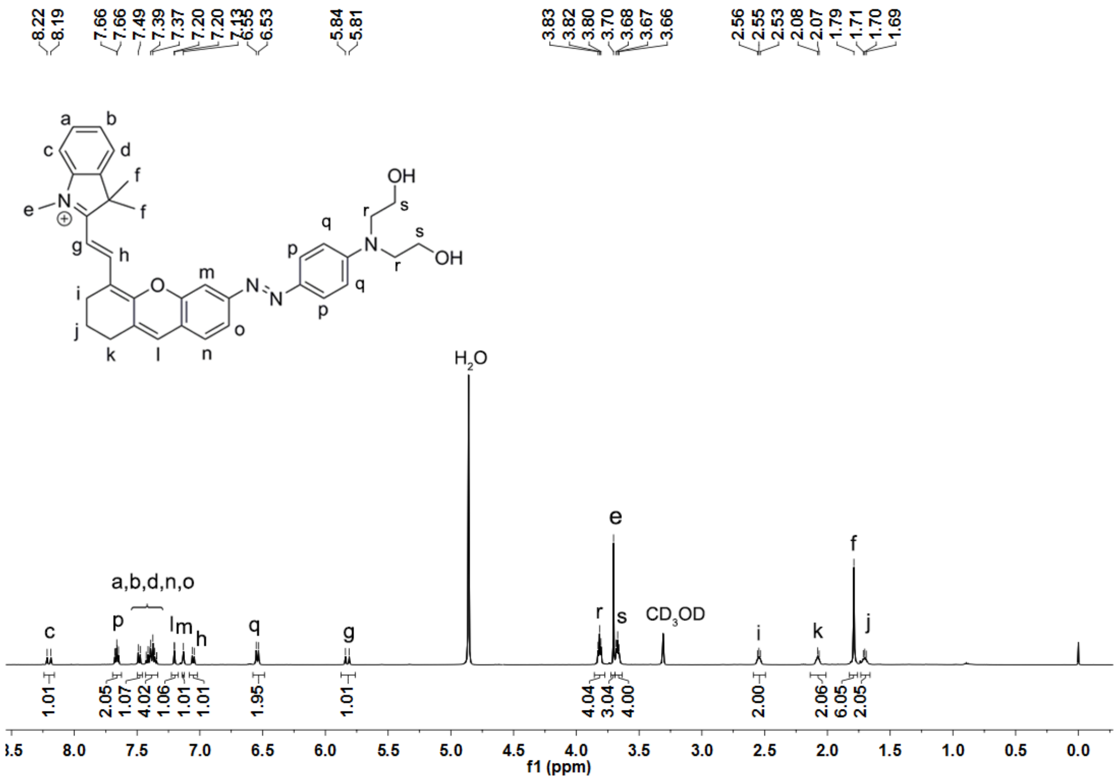

Figure S4. ^{1}H NMR spectrum of compound 3 (in CD_3OD).

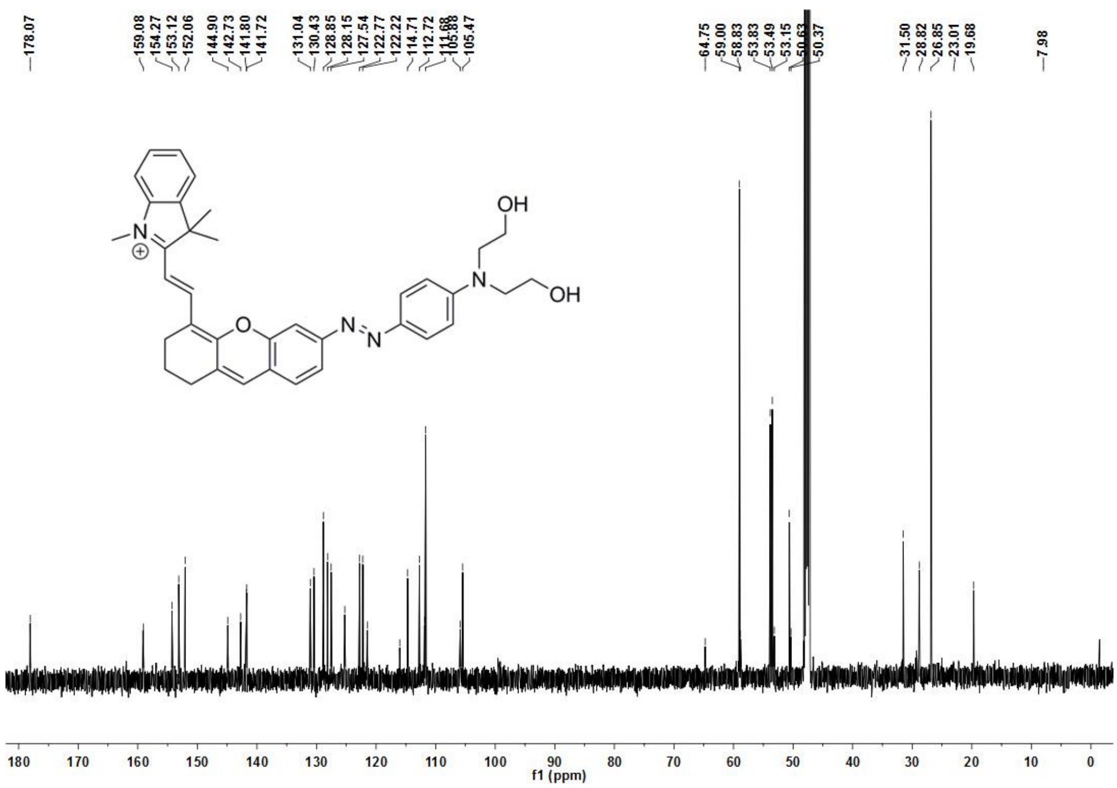

Figure S5. ^{13}C NMR spectrum of compound 3 (in CD_3OD).

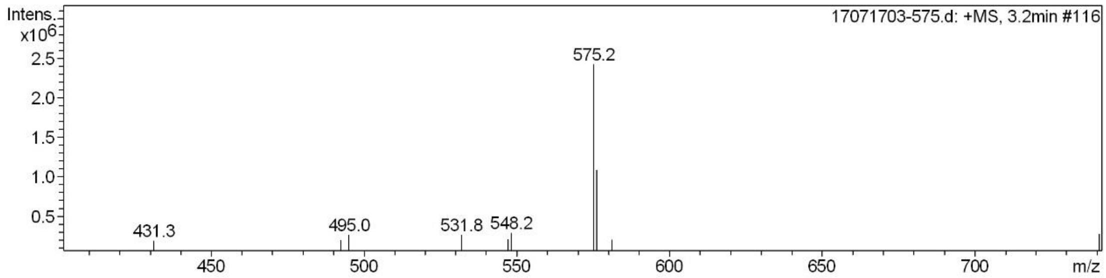

Figure S6. MS spectrum of compound 3.

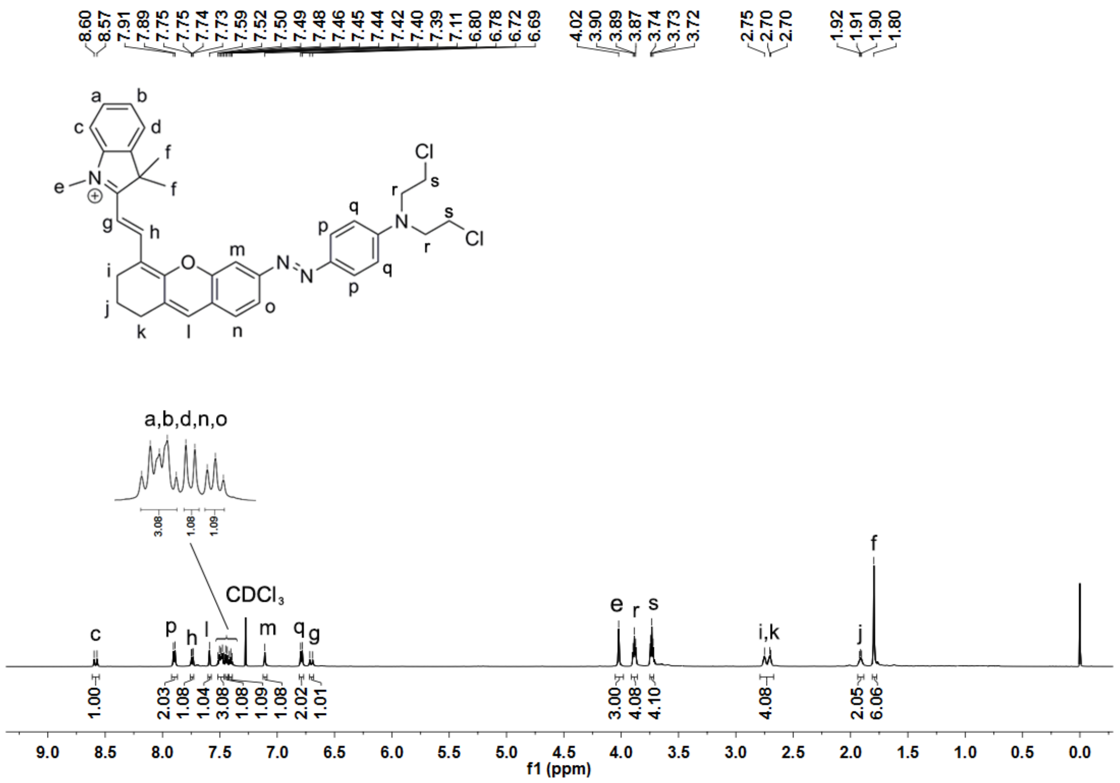

Figure S7. ^{1}H NMR spectrum of compound 4 (in $CDCl_3$).

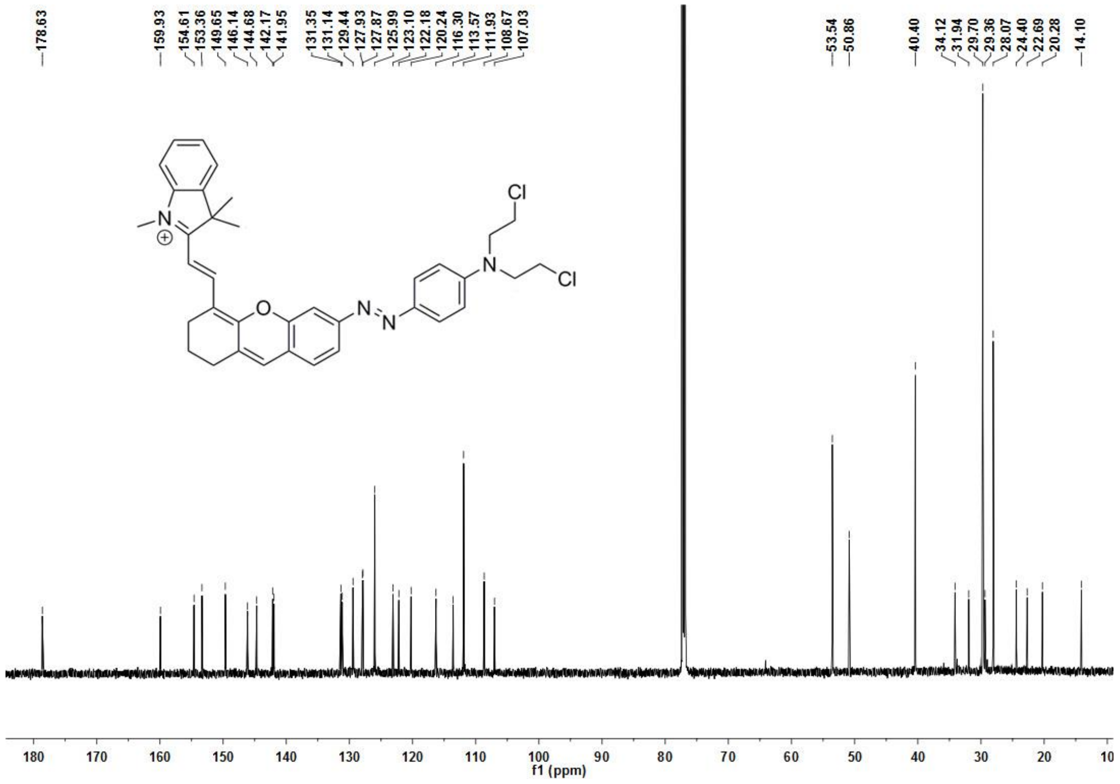

Figure S8. ^{13}C NMR spectrum of compound 4 (in $CDCl_3$).

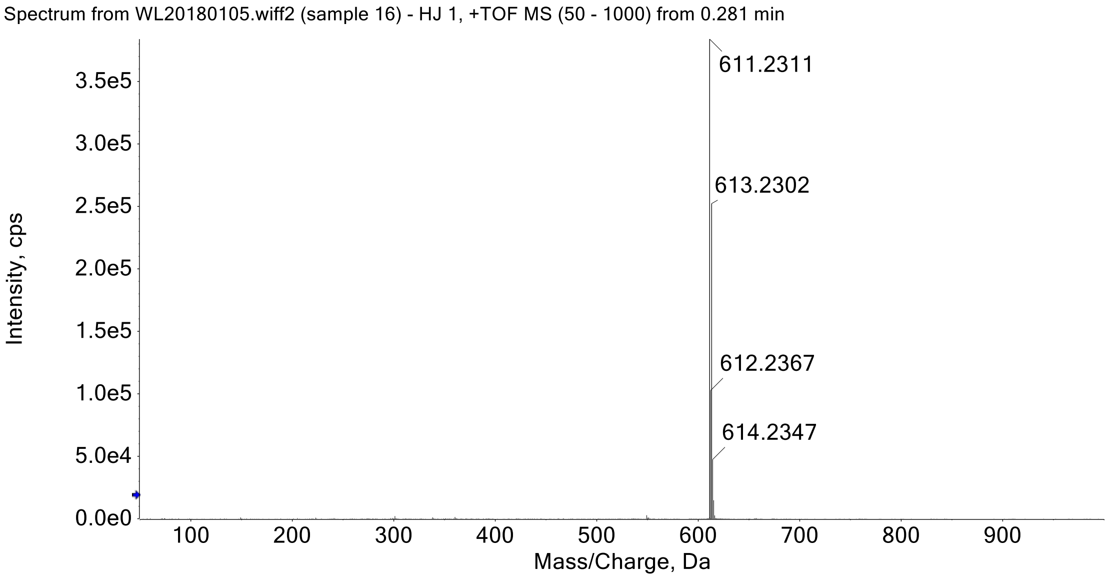

Figure S9. HR-MS spectrum of compound 4.

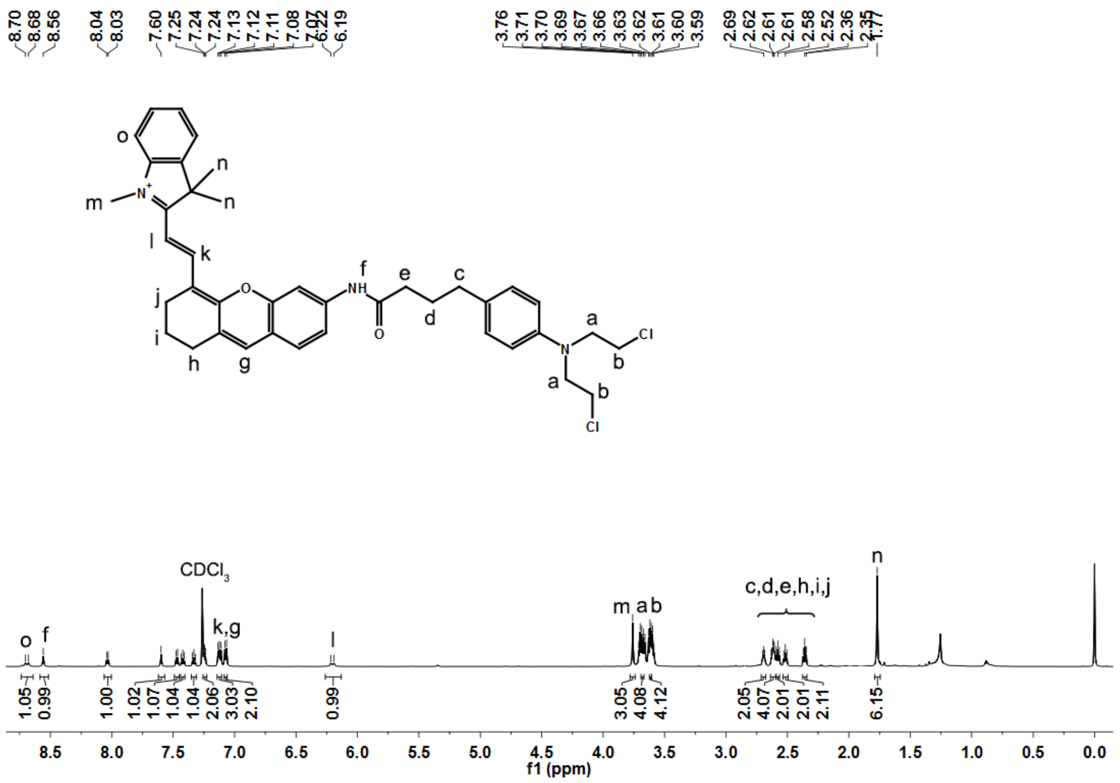

Figure S10. ^{1}H NMR spectrum of control chromophore (in $CDCl_3$).

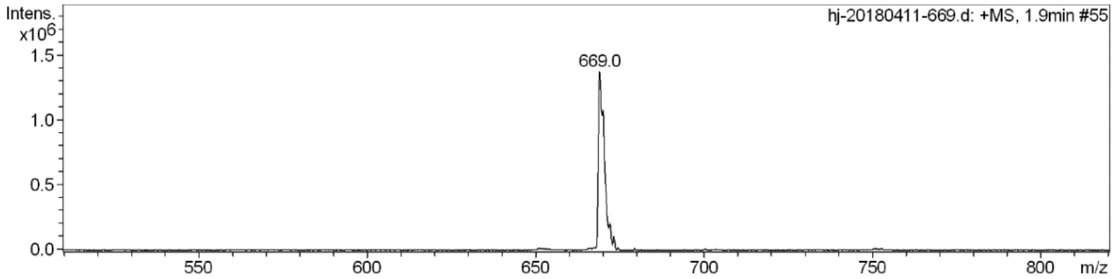

Figure S11. MS spectrum of control chromophore.

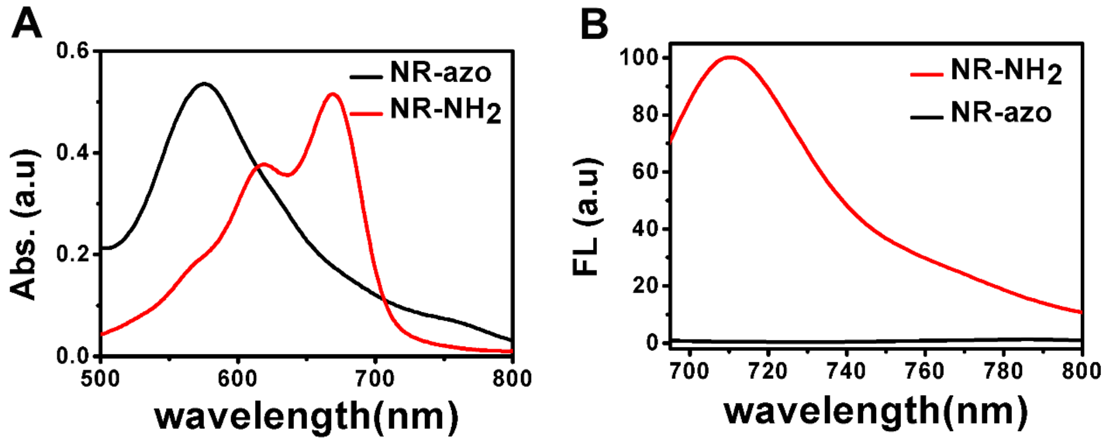

Figure S12. A) Absorption spectra of NR-azo (10 μM) and NR-NH_2 (10 μM). B) Fluorescence spectra of NR-azo (10 μM) and NR-NH_2 (10 μM). λex = 680 nm Optical properties were examined in phosphate-buffered saline (10 mM PBS, pH=7.4) containing 1% DMSO.

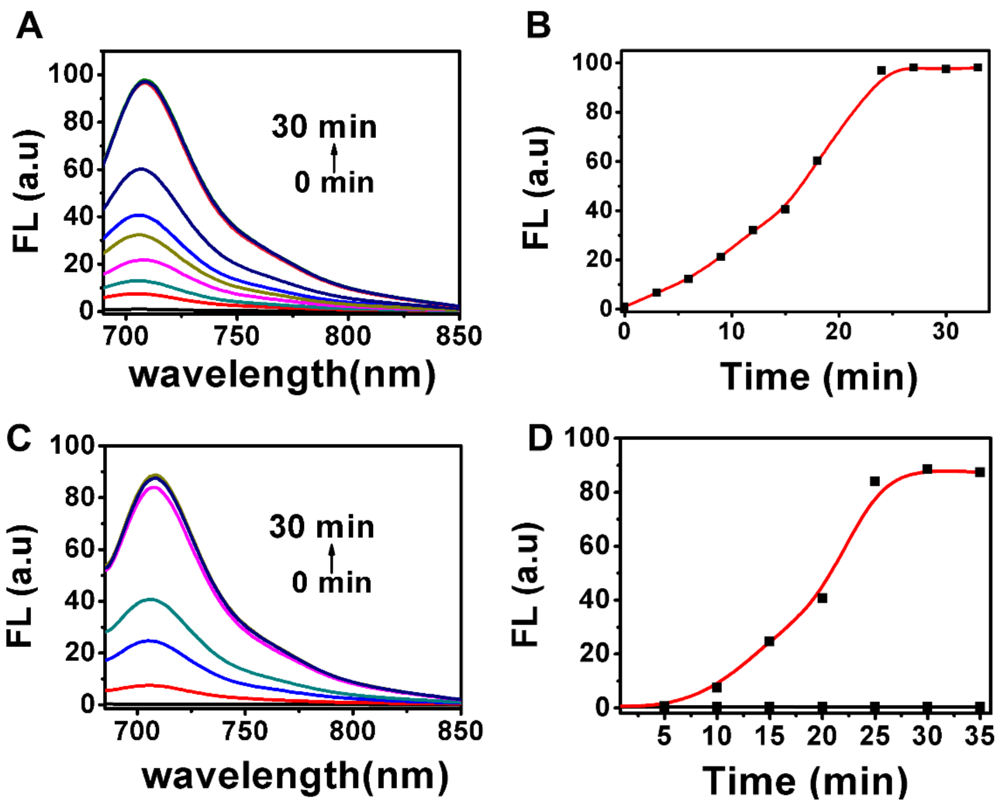

Figure S13. A) Time-dependent fluorescence spectra of NR-azo (10 μM) upon addition of SDT measured in deionized water under normal atmosphere. B) Fluorescence intensity at 707 nm with the treatment of SDT under normal atmosphere. C) Time-dependent fluorescence spectra of NR-azo (10 μM) upon addition of MLM (50 μg/mL) under nitrogen atmosphere, measured in phosphate-buffered saline (10 mM PBS, pH=7.4, containing 1% DMSO, with 100 μM NADPH). D) Fluorescence intensity at 707 nm upon the treatment with MLM and NADPH under nitrogen atmosphere (red curve) and under normal atmosphere (black curve). λex = 680 nm

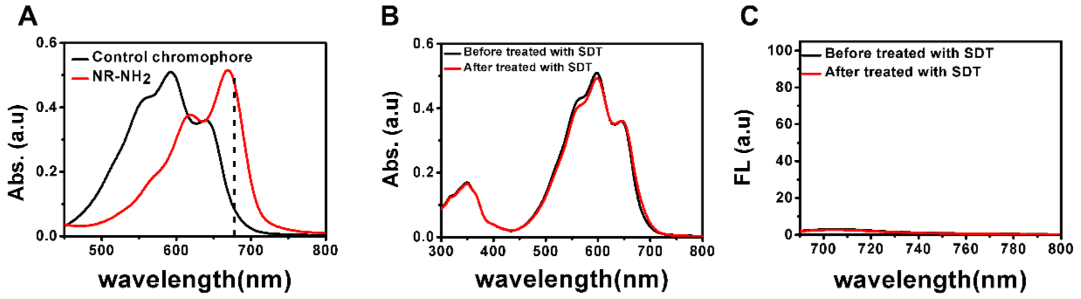

Figure S14. A) Absorption spectra of the control chromophore (10 μM) and NR-NH_2 (10 μM) measured in PBS (10 mM, pH 7.4, containing 1% DMSO). B) Absorption spectra of the control chromophore before and after treatment with SDT. C) Fluorescence spectra of the control chromophore before and after SDT treatment. λex = 680 nm.

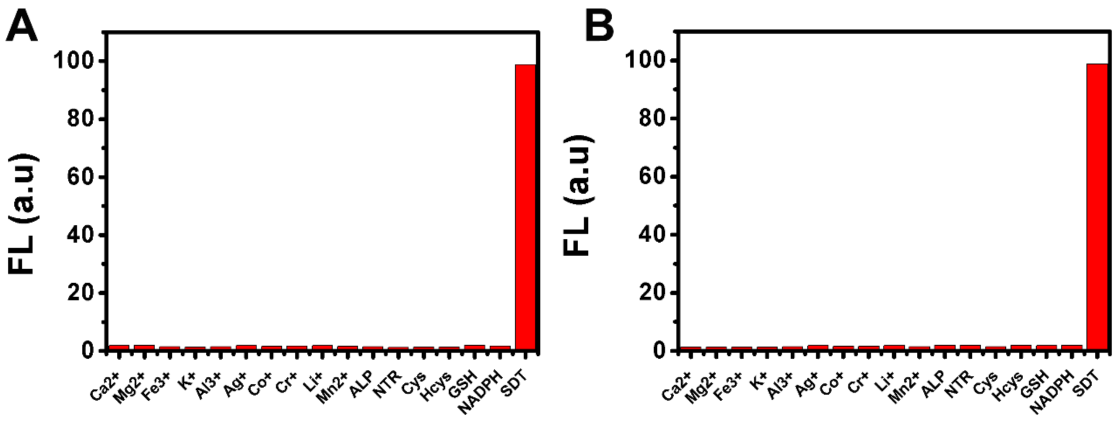

Figure S15. Selectivity experiment under nitrogen atmosphere A) and normal atmosphere B). NR-azo was treated with SDT (100 μM) or various substances: $MgCl_2$ (1 mM), $CaCl_2$ (1 mM), $FeCl_3$ (1 mM), KCl (1 mM), $AlCl_3$ (1 mM), $AgNO_3$ (1 mM), $CoCl_2$ (1 mM), $CrCl_3$ (1 mM), LiCl (1 mM), $MnCl_2$ (1 mM), ALP (100 U/L), NTR (10 μg/mL), Cys (100 μM), Hcy (100 μM), GSH (100 μM), NADPH (100 μM) under normal atmosphere and nitrogen atmosphere at 37 ℃. Incubation time 30 minutes. λex = 680 nm.

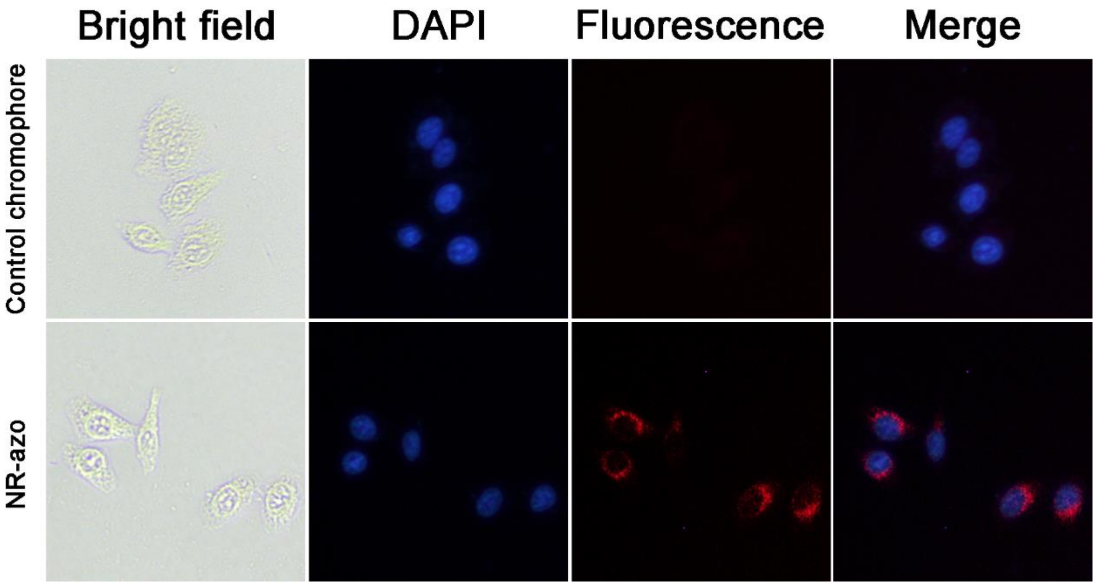

Figure S16. Fluorescence images for HepG2 cells incubated with 10 μM NR-azo or 10 μM control chromophore under hypoxia for 6 h. Excitation filter: 675 nm. (DAPI was used for nuclei staining)

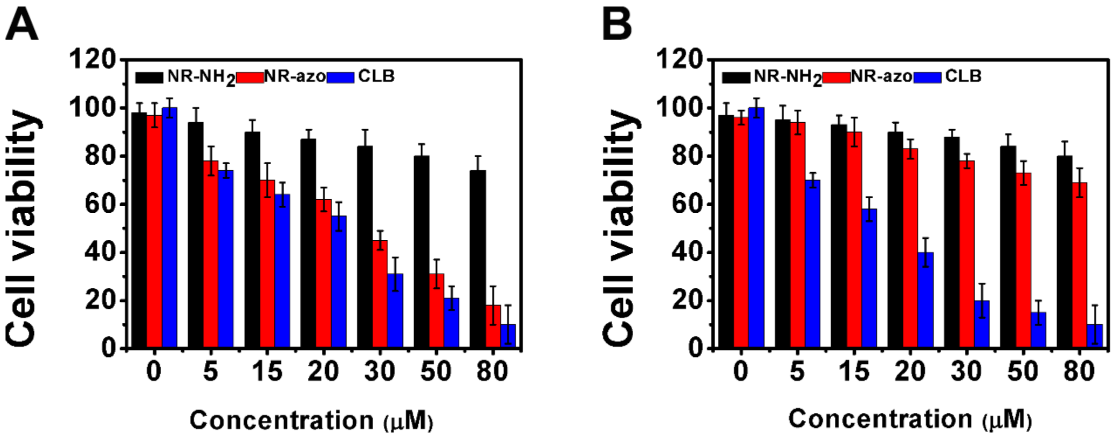

Figure S17. MTT experiment under hypoxia (A) and normoxia (B).

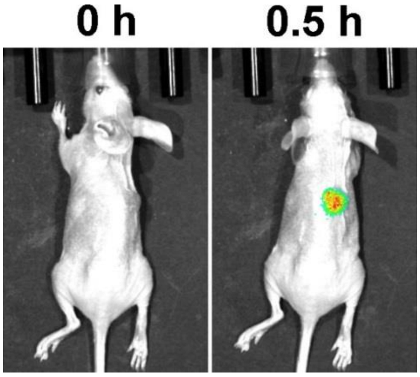

Figure S18. In vivo fluorescence imaging of mouse intratumorally injected with NR-NH_2 (imaging was performed at 0.5 h post injection).

At 0.5 h post intratumoral injection of NR-NH_2, the NR-NH_2 mainly remained in tumor site, which indicates the diffusion to other tissues is relatively slow.

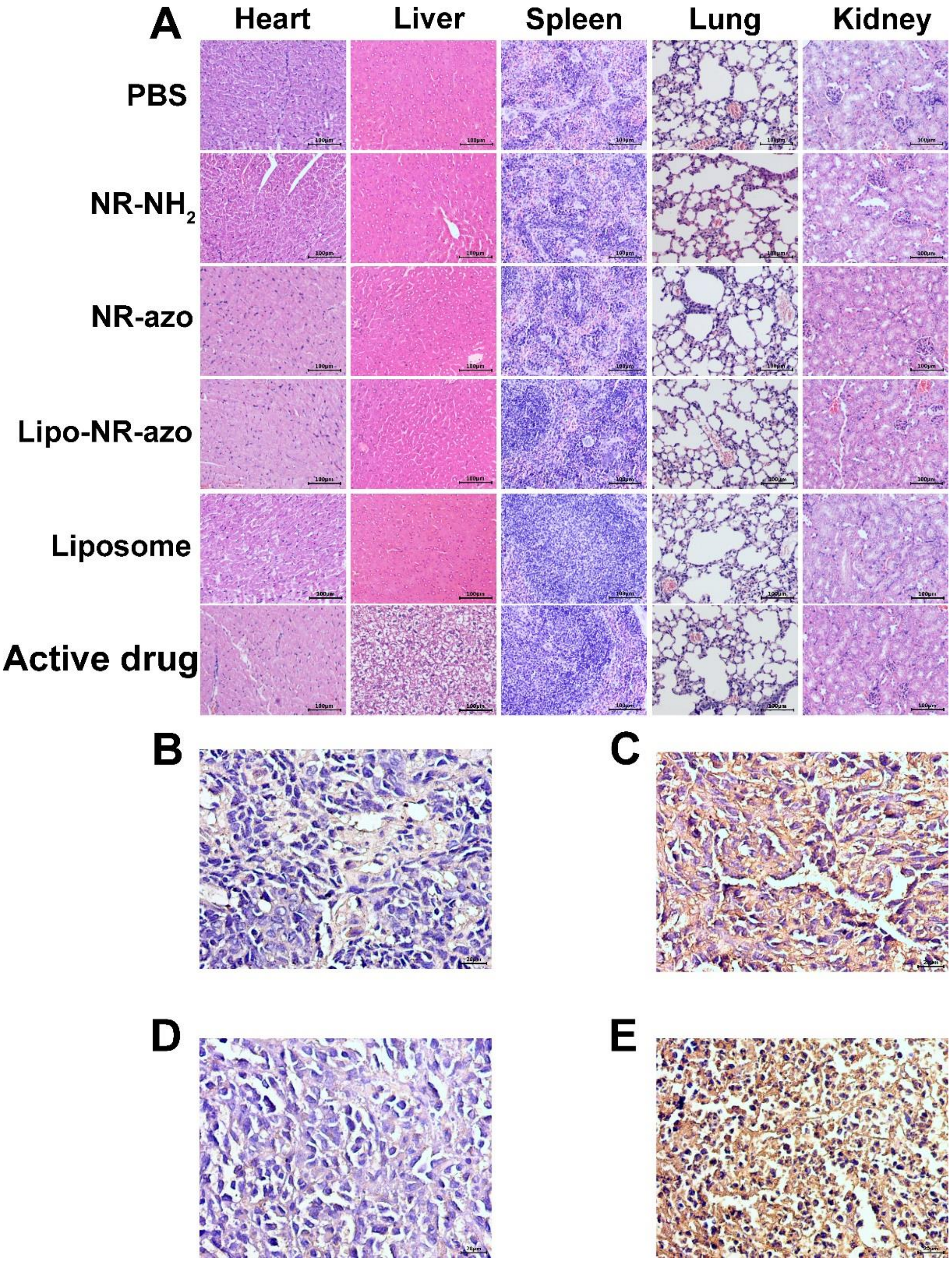

Figure S19. A) H&E staining analysis of tissues from main organs of the healthy mice groups treated with different formulations for 24 h. Scale bar: 100 μm. B) and C) Immunohistochemical analysis of CD206 (B: tissue at the left oxter from a healthy mouse; C: tumor tissue at the oxter from a tumor-bearing mouse). The brown color indicates the high expression of CD206. Scale bar: 20 μm. D) and E) Representative TUNEL staining of tumor sections for the mice upon treated with PBS (D) and Lipo-NR-azo (E), the brown color indicates the apoptosis of cells. Scale bar: 20 μm.

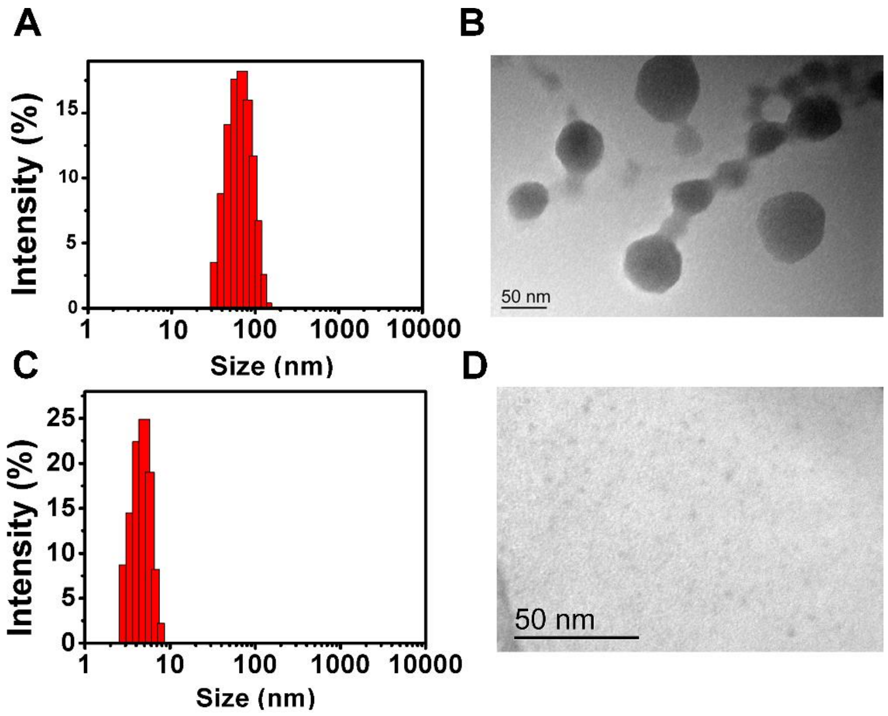

Figure S20. A) Particle size distribution of Lipo-NR-azo measured by DLS. B) TEM images of Lipo-NR-azo particles. C) Particle size distribution of NR-azo measured by DLS. D) TEM images of NR-azo.

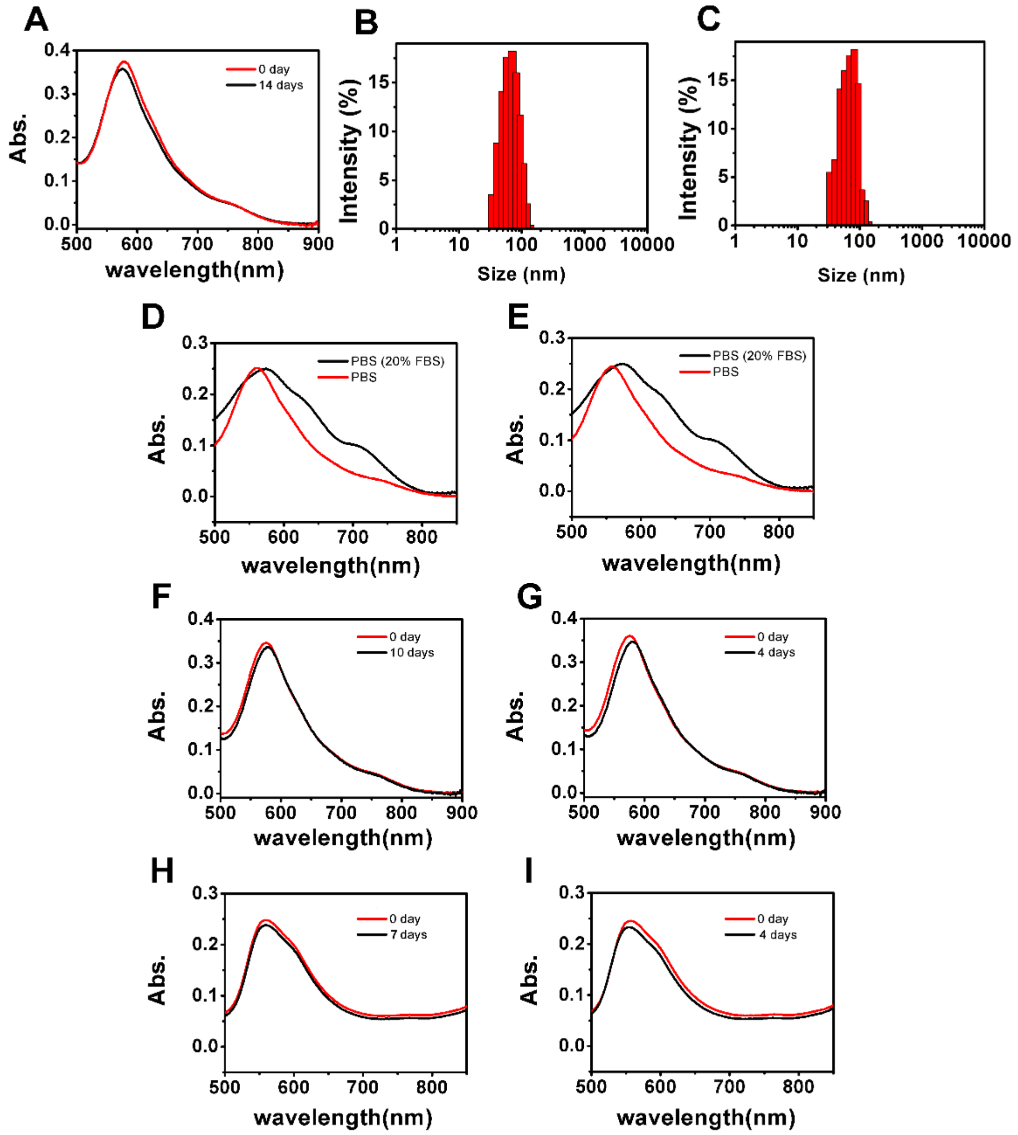

Figure S21. Stability of Lipo-NR-azo and NR-azo. A) Absorption spectra of Lipo-NR-azo before (red line) and after (black line) storage at 4 ℃ for 14 days. B,C) Size distribution of Lipo-NR-azo measured by DLS before (B) and after (C) storage at 4 ℃ for 14 days. d,e) Absorption spectra of NR-azo before (D) and after (E) storage for 14 days (red line: dissolved in PBS at 25 ℃, black line: dissolved in PBS that containing 20% Fetal Bovine Serum at 4 ℃). F,G) Absorption spectra of Lipo-NR-azo (dissolved in PBS) under the storage at 25 ℃ (F) and 37 ℃ (G) for different days. H,I) Absorption spectra of NR-azo (dissolved in PBS that containing 20% Fetal Bovine Serum) under the storage at 25 ℃ (H) and 37 ℃ (I) for different days.

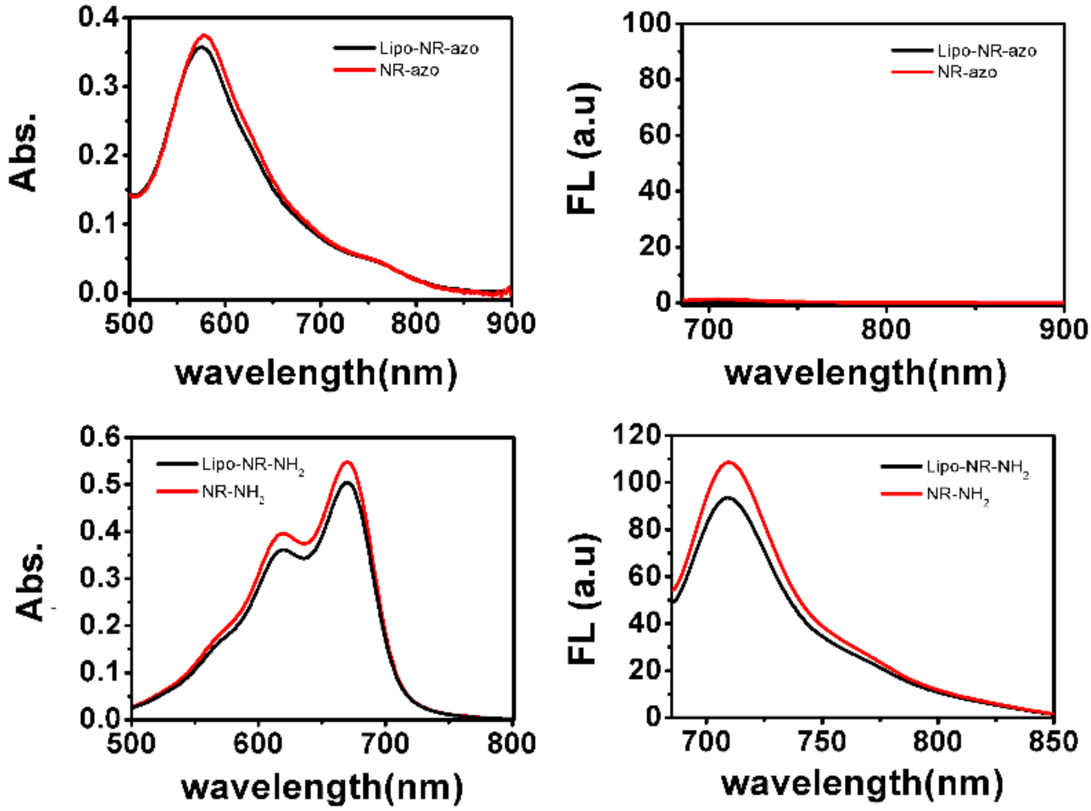

Figure S22. Spectral properties of Lipo-NR-azo, Lipo-NR-NH_2, NR-azo and NR-NH_2.

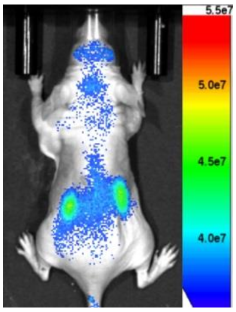

Figure S23. In vivo fluorescence imaging of the xenograft tumor-bearing mouse with intravenous injection of NR-NH_2 (in PBS containing 1 % DMSO, 10 mM, pH 7.4), at 1 h post-injection. The mouse lay on its stomach during imaging, and the fluorescent signals mainly reside in the kidney region.

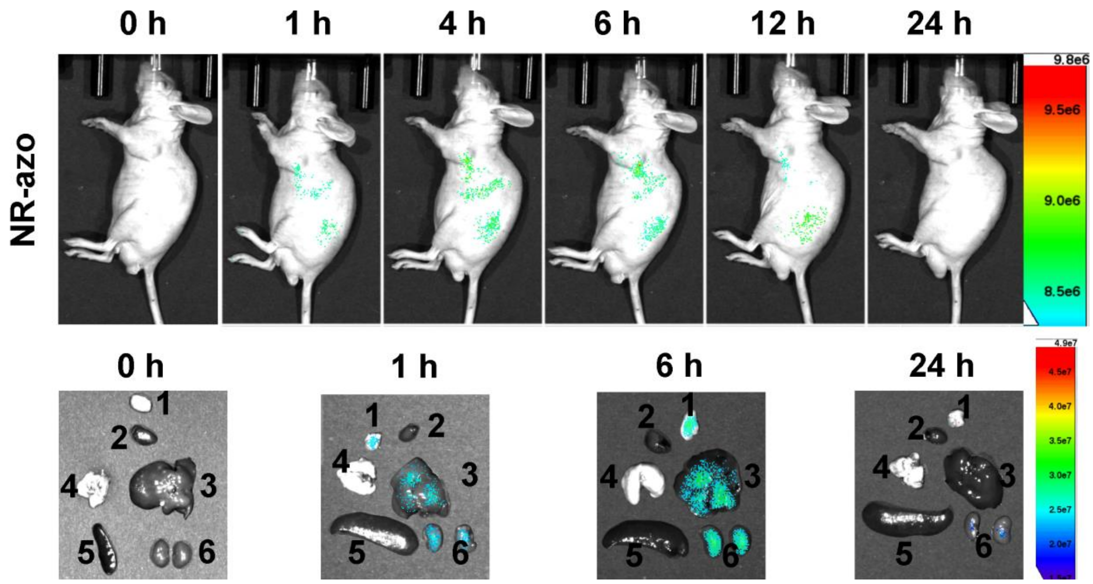

Figure S24. Upper row: Typical fluorescence images of the xenograft tumor-bearing mice upon intravenous injection of NR-azo (in PBS containing 1 % DMSO) for different time.

Lower row: Ex vivo fluorescence images of major organs and tumors collected from the tumor-bearing mice sacrificed at different time points after treatment with NR-azo (1: tumor, 2: heart, 3: liver, 4: lung, 5: spleen, 6: kidneys).

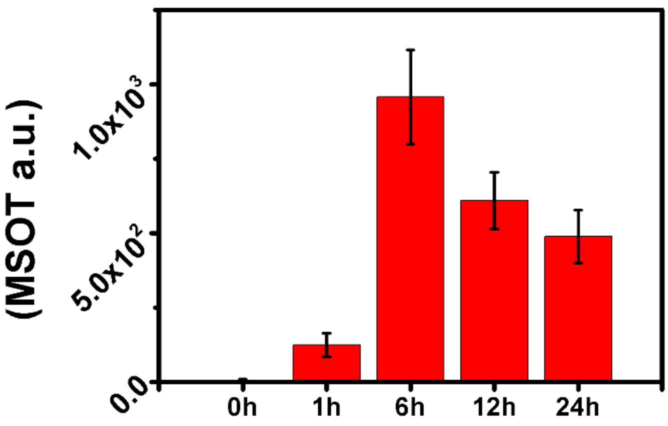

Figure S25. The MSOT intensities at tumor site of the tumor-bearing mouse at varied time upon intravenous injection of Lipo-NR-azo (in PBS).

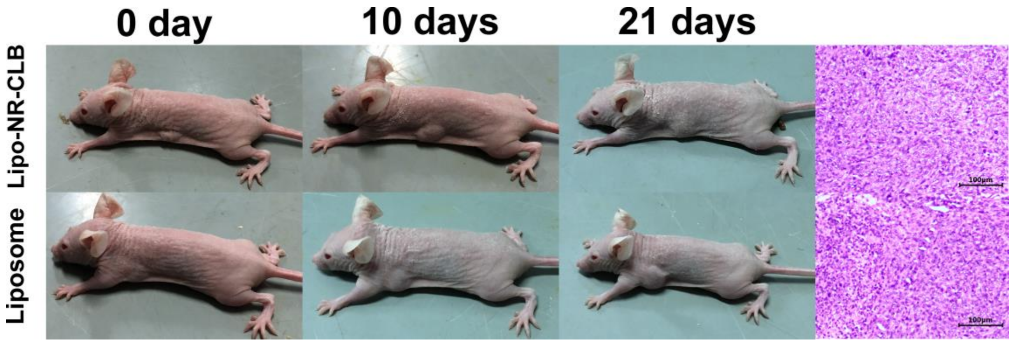

Figure S26. Photographs of live mice treated with different formulations during the 21-day period. H&E staining analysis of tissues from the tumors of the mice treated with Lipo-NR-CLB and Liposome respectively at day 21 (Scale bar: 100 μm).

For the mice treated with active drug, no mice survived more than 1 week.

References:

1. Wu Y, Huang S, Wang J, Sun L, Zeng F, Wu S. Activatable probes for diagnosing and positioning liver injury and metastatic tumors by multispectral optoacoustic tomography. Nat Commun. 2018; 9: 3983.
2. Piao W, Tsuda S, Tanaka Y, Maeda S, Liu F, Takahashi S, et al. Development of azo-based fluorescent probes to detect different levels of hypoxia. Angew Chem Int Ed Engl. 2013; 52: 13028-13032.
3. Xu KH, Wang F, Pan XH, Liu RP, Ma J, Kong FP, et al. High selectivity imaging of nitroreductase using a near-infrared fluorescence probe in hypoxic tumor. Chem Commun (Camb). 2013; 49: 2554-6.
4. Martin CN, Kennelly JC. Rat liver microsomal azoreductase activity on four azo dyes derived from benzidine, 3,3'-dimethylbenzidine or 3,3'-dimethoxybenzidine. Carcinogenesis. 1981; 2: 307-12.
